# Dynamic reconfiguration of macaque brain networks during natural vision

**DOI:** 10.1016/j.neuroimage.2021.118615

**Published:** 2021-12-01

**Authors:** Michael Ortiz-Rios, Fabien Balezeau, Marcus Haag, Michael C. Schmid, Marcus Kaiser

**Affiliations:** a Bioscience Institute, Henry Welcome Building, Medical School, Framlington Place, Newcastle upon Tyne NE2 4HH, UK; b Functional Imaging Laboratory, Deutsches Primatenzentrum (DPZ), Leibniz-Institut für Primatenforschung, Göttingen, Germany; c School of Computing, Urban Sciences Building, Newcastle University, Science Central, Newcastle upon Tyne NE4 5TG, UK; d Precision Imaging Beacon, School of Medicine, University of Nottingham, UK; e Shanghai Jiao Tong University, Rui Jin Hospital, Department of Functional Neurosurgery, China; f Faculty of Science and Medicine, University of Fribourg, Chemin du Musée 5, 1700 Fribourg, Switzerland

**Keywords:** Macaque monkey, fMRI, BOLD, Free-viewing networks, Structural networks

## Abstract

Natural vision engages a wide range of higher-level regions that integrate visual information over the large-scale brain network. How interareal connectivity reconfigures during the processing of ongoing natural visual scenes and how these dynamic functional changes relate to the underlaying anatomical links between regions is not well understood. Here, we hypothesized that macaque visual brain regions are poly-functional sharing the capacity to change their configuration state depending on the nature of visual input. To address this hypothesis, we reconstructed networks from *in-vivo* diffusion-weighted imaging (DWI) and functional magnetic resonance imaging (fMRI) data obtained in four alert macaque monkeys viewing naturalistic movie scenes. At first, we characterized network properties and found greater interhemispheric density and greater inter-subject variability in free-viewing networks as compared to structural networks. From the structural connectivity, we then captured modules on which we identified hubs during free-viewing that formed a widespread visuo-saccadic network across frontal (FEF, 46v), parietal (LIP, Tpt), and occipitotemporal modules (MT, V4, TEm), and that excluded primary visual cortex. Inter-subject variability of well-connected hubs reflected subject-specific configurations that largely recruited occipito-parietal and frontal modules. Across the cerebral hemispheres, free-viewing networks showed higher correlations among long-distance brain regions as compared to structural networks. From these findings, we hypothesized that long-distance interareal connectivity could reconfigure depending on the ongoing changes in visual scenes. Testing this hypothesis by applying temporally resolved functional connectivity we observed that many structurally defined areas (such as areas V4, MT/MST and LIP) were poly-functional as they were recruited as hub members of multiple network states that changed during the presentation of scenes containing objects, motion, faces, and actions. We suggest that functional flexibility in macaque macroscale brain networks is required for the efficient interareal communication during active natural vision. To further promote the use of naturalistic free-viewing paradigms and increase the development of macaque neuroimaging resources, we share our datasets in the PRIME-DE consortium.

## 1 Introduction

During natural vision, the brain is in a high dynamical state generating perception and action from the collective interactions among widespread brain areas. Interareal interactions can be studied by measuring the temporal correlation between BOLD signals of any pair of brain areas. These interactions are known to fluctuate between states of high and low connectivity strength over time and relate to patterns of synchrony over the macroscale brain network (Zalesky et al., 2014). How these state fluctuations relate to internal or external triggers for reconfiguration remains challenging to infer via resting-state paradigms and we lack fundamental knowledge about how the brain reconfigures during dynamic natural vision.

Recently, an increasing number of studies, in both human and monkeys, began to implement naturalistic viewing paradigms for mapping whole-brain activity with rich temporal dynamics (Bartels and Zeki, 2004; Bartels et al., 2008; Hung et al., 2015; McMahon et al., 2015; Russ and Leopold, 2015; Russ et al., 2016). In humans, studies using the naturalistic free-viewing paradigm demonstrated the high degree of similarity between activation maps obtained during movie viewing and maps obtained with controlled fixation (Bartels and Zeki, 2004; Bartels et al., 2008). Additional studies demonstrated how functional connectivity patterns were synchronized across individuals watching the same movie scenes (Hasson et al., 2004; Lahnakoski et al., 2012), further highlighting the reliability and effectiveness of the approach.

Comparative research between humans’ and macaques’ watching the same movies identified homologous and divergent brain regions across primate species (Betti et al., 2013; Mantini et al., 2012), with more detailed mapping revealing a previously unknown predominance of visual motion in areas well-described for face processing in the macaque (Russ and Leopold, 2015; Russ et al., 2016), and similarly in the human brain (Hasson et al., 2004). Moreover, recent studies (Russ and Leopold, 2015; Sliwa and Freiwald, 2017) highlighted how the free-viewing paradigm can be used for mapping specialized functional regions of the inferotemporal cortex and beyond.

**Fig. 1:**
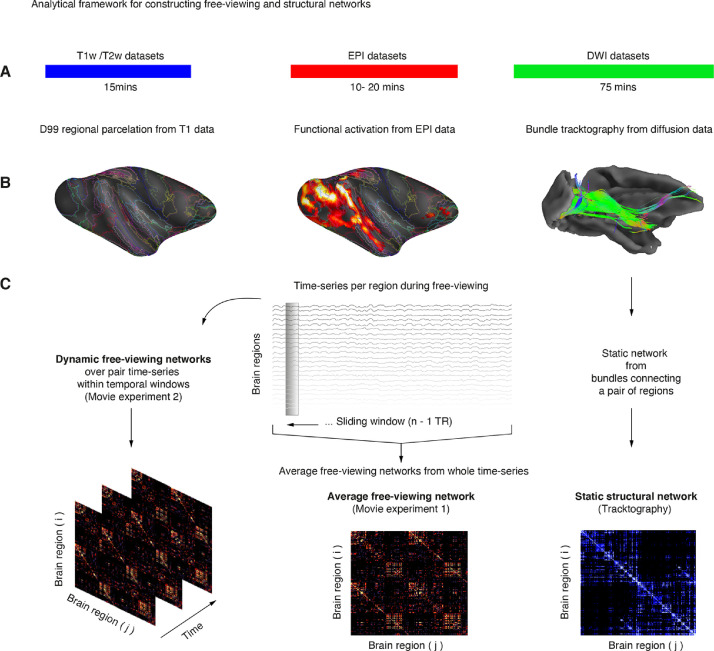
**Analytical framework for constructing free-viewing and structural networks. A**. Workflow for the acquisition of structural T1, T2, DWI, and fMRI datasets. All data was approximately acquired within less than two hours of scanning time (see **Supplementary Fig. 1** for the complete pipeline). **B**. Preliminary assessment of the quality of the dataset prior to construction of structural and functional networks. The NHP-specific parcellation was obtained from the D99 atlas (left), while functional data was initially assessed via standard GLM analysis (middle). The bundle tractography of the visual cortex was also assessed prior to the construction of structural networks as a strategy to evaluate the quality of diffusion data obtained during the awake state (right, see **Supplementary Fig. 6** and **Supplementary Fig. 7** for example subject data). **C**. Following preliminary analyses, we then build three types of networks: Dynamic free-viewing networks, static-free viewing networks, and structural networks. Dynamic free-viewing networks were obtained over 18‑*sec* temporal window partitions, while average free-viewing networks were obtained over the whole time series of regional pairs (*i)* and (*j)*. Structural networks were obtained from the number of streamlines (*n >* 10) that connected any pair of regions (*i)* and (*j)*.

In this study, we build subject-specific macaque brain networks that rely on the interareal interactions present during movie watching. From the recent network studies in humans demonstrating the reconfiguration of inferotemporal networks during the viewing of faces (Rosenthal et al., 2017) and from the recent free-viewing studies in NHPs reviewed above we hypothesized that macaque brain networks– including those from the inferotemporal cortex–are more poly-functional in their representational state than previously conveyed with trial-by-trial designs. To capture dynamical changes in networks we evaluated time-varying functional connectivity using a sliding window across the time-series (Fig. 1). Prior to assessing dynamic functional connectivity, we first captured the static state of the network via diffusion tractography (Bassett and Sporns, 2017; Bullmore and Sporns, 2009). Furthermore, we characterized networks and identified a widespread visuo-saccadic network with central hubs connecting over long-range distances. From these results we hypothesized that long-distance hub regions could reconfigure depending on the ongoing changes in visual scenes. Departing from the static structure and via time-varying functional connectivity we captured dynamical changes in interareal interactions across cortico-cortical and thalamocortical areas during each main scene. In the discussion, we relate our findings with the well-known functional and anatomical properties of the macaque visual system and discuss how natural-viewing paradigms in NHPs combined with network neuroscience can provide new insights into the nature of macroscale cognitive systems during active natural vision.

## 2 Materials and methods

### 2.1 Subjects

Four female rhesus monkeys (*Macaca mulatta*) were used to obtain all neuroimaging data; (VL, six years of age, weighing 7 kg; DP, six years of age, weighing 9 kg; FL 4 years of age, weighting 6 kg; AL, five years of age, weighing 9 kg). All animals were socially housed in an environmentally enriched home cage.

An essential facet of NHP neuroimaging, as opposed to human neuroimaging, is the requirement for head stabilization during data acquisition. Head stabilization in NHPs requires head post implants. Crucially, the size and material of implants affect the quality of echo-planar images near the implantation region, which further affects the reliability and construction of whole-brain networks from imaging data. Here, we applied a customized head-post implant procedure and obtained distortion-free MR images of the entire brain. All surgical and anesthesia procedures, postoperative care, and implant methods were described in previous work (Ortiz-Rios et al., 2018).

The UK Home Office approved all procedures, complying with the Animal Scientific Procedures Act (1986) for the care and use of animals in research and the European Directive on protecting animals used in research (2010/63/EU).

### 2.2 Awake macaque neuroimaging

Before data acquisition, we trained monkeys to voluntarily approach the inside of a wooden box from which they learned to enter a cylindrical MRI-compatible chair. Using positive reinforcement techniques, we trained animals to lift their heads outside of the chair and to remain calm in the chair.

Once habituated, we exposed the animals to the scanner environment and trained them to remain calm. Acclimatization within the scanner took one month to accomplish. Once animals were implanted and comfortable with the procedure, their heads were immobilized to the MRI chair using a PEEK head holder after two months post-implantation. We protected the monkey's hearing from the scanner noise by using standard ear muffs. During the scanning period, animals remained calm while watching five minutes movie clips. Typically, while being trained or scanned in the absence of any visual stimulation, in darkness, or during anatomical scanning, animals remained calm or in a sleep-relaxed condition. Acclimatizing the macaques for awake imaging took approximately three months to accomplish.

#### 2.2.1 Stimuli presentation

A 45-degree angled back-projection mirror system was used to display all movie stimuli. A projector (NEC NP1150) displayed images onto a screen (size: 35 length × 32 width cm) placed at a distance of 73.5 cm from the center of the monkey's head. The projection created a visual field of 26 × 24° of visual angle.

An infrared-based eye-tracking system (iView, SensoMotoric Instruments GmbH, Teltow, Germany) received video signals from an MRI-compatible camera (12M-I with integrated LED, MRC Systems, GmbH) placed behind a mirror in front of the animal's eye. An analog-to-digital conversion card (NI-USB 6212- BNC, National Instruments) then sampled the analog signal from the eye-tracker. Additionally, we used the digital-to-analog output from the NI-card to control the reward system and trigger data acquisition. MWorks software (https://mworks.github.io) installed on a MacMini computer (2.8 GHz, Core i5) managed the NI-card for controlling all peripheral devices.

#### 2.2.2 Movie experiment one

The initial movie clips contained unfamiliar visual scenes with ego-perspective camera motion. For example, the movie clips showed an ego perspective of a human driving a bike across rough terrain (https://zenodo.org/record/5026036#.YTINgC2Q2uU. The following two segments showed two human individuals climbing a transmission power tower. We presented five movie clips (30 sec on and OFF) lasting a total of five minutes within an imaging run. During the OFF period, the screen remained dark. To avoid jaw movements, we opted for no reward delivery during the scan. Instead, we delivered a juice reward to the animal after the movie and acquisition period ended. For comparisons, the movie content was kept constant across repetitions and sessions, allowing us to relate connectivity patterns across all subjects.

#### 2.2.3 Movie experiment two

For the second series of free-viewing experiments, each movie segment lasted 30 s, followed by a dark interval period of 15 s. We presented twenty-five movie segments in a run which lasted ∼20 min. We modified each movie category to control for low-level visual features on a frame-by-frame basis. For the phase scrambling, the original frames were Fourier transformed using Matlab. The phase was then randomized and added to the initial phase. We calculated the inverse Fourier over the transformed data to generate an image. For the additional controls, we used the Matlab functions *randblock* for tile scrambling, the *spectral visual saliency toolbox* to create saliency contour images, and the *OpticalFlow Matlab* function to create vector motion direction on each frame.

### 2.3 Multimodal data acquisition

We used a 4.7 Tesla vertical magnet running ParaVision 5.1 (Bruker, BioSpin GmbH, Ettlingen, Germany) and equipped with a 4-channel phase-array coil (https://www.wkscientific.com) for data acquisition. All neuroimaging data was based on three sequence types: 1) Diffusion-weighted imaging (DWI) for tractography, 2) echo-planar imaging (EPI) for functional imaging, and 3) 3D-modified driven equilibrium Fourier transform (MDEFT) for anatomical imaging (see **Supplementary Table 1**).

### 2.4 Overall pipeline workflow for the analyses of multimodal imaging data

We illustrate the complete workflow of data pre-processing and analyses for constructing networks in **Supplementary Fig. 1**. The pipeline consisted of two pre-processing steps for each dataset type: T1 (blue in steps 1–4), DWI (green 5–7), and EPI (red in steps 8–10). The final output of the pre-processing pipeline provided structural (magenta in step 7) and free-viewing networks (pink in step 11). All pre-processing analyses were performed using open software provided in the packages of AFNI (Cox, 1996), SUMA (Saad et al., 2004) and Freesurfer (Dale et al., 1999). Connectivity and network measures were performed using the Brain Connectivity Toolbox (BCT) (https://sites.google.com/site/bctnet/Home and the Network analysis and visualization toolbox in Matlab https://uk.mathworks.com/help/bioinfo/network-analysis-and-visualization-1.html).

### 2.5 Anatomical data pre-processing and atlas parcellation

The in-session T1 volume was warped into the D99 (Reveley et al., 2017) atlas generating a surrogate brain. We used the *align_macaque_script* (Step 1) to generate all atlas files. We then optimize small ROIs for network construction. For white matter segmentation (Step 2), we used the command

*3dSeg -anat “input.nii” -mask AUTO -classes output.nii -bias_classes “output.ni” -bias_fwhm 25 -mixfrac UNI -main_N 5 -blur_meth BFT,* to obtain a white matter (WM) mask. The output from the *align_macaque_script* included the atlas ROIs parcellation of the D99 template (Step 3). To segment the cortical gray matter, we binarized the D99 atlas using the programs *3dAutomask* and *3dcalc* (Step 4). To generate a rendered white, pial (Step 5), and inflated surfaces (Step 6), we provided the masks to FreeSurfer for rendering. For displaying the ROIs on the brain surface, we first created a color mappable .niml file of the atlas volume using the command,

*3dVol2Surf -spec input.spec -surf_A lh.surface.gii -sv input.nii -grid_parent rois.nii -use_norms -norm_len 2.5 -map_func* max *-f_steps 10 -f_index nodes -out_niml Lh.output.niml.dset.*

We then display the activation results in SUMA along with ROIs contours.

### 2.6 fMRI data pre-processing

fMRI data was pre-processed using (Analyses of functional neuroimages–AFNI). Time series preprocessing (Step 8) included: slice-timing correction, motion correction, spatial smoothing, and normalization (a voxel-by-voxel scaling of the time series by the mean). Slice-timing differences were corrected from the fMRI time-series using the command

*3dTshift -tzero 0 -prefix “output.nii” “input.nii”*.

The potential occurrence of spikes–transient artifacts often caused by small electrical discharges or movement–were detected using the command,

*3dDespike -NEW25 -localedit -prefix “output.nii” “input.nii”*.

For despiking, we fitted a linear regression curve into the time series and calculated the mean absolute deviation (MAD) of the residuals. The fraction of outliers was then calculated from the time series using the command,

*3dToutcount -automask “input.nii” > “output.1D”*.

Next, we performed motion correction by first calculating a mean baseline volume using the command,

*3dTstat -prefix “epi.mean.nii” “input.nii”*.

We then proceeded with motion correction with the command,

*3dvolreg -verbose -base “epi.mean.nii” -dfile “output.1D” -prefix “output.nii” “input.nii”.*

*3dvolreg* applied a rigid body transformation with six motion parameters (three translations and three rotations) to each time-point in the time series to match the voxelwise mean. The output of *3dvorleg* provided motion parameters (output.1D file) which were used in the regression analyses as nuances of no-interest. Using the python script,

*1d_tool.py -infile input.1D -set_nruns 1 -show_censor_count -overwrite -censor_motion 0.5 “output.1D”*, we detected motion shifts greater than 0.5 mm and rotations greater than 0.5 °. The output time-series included more than 98% of the volumes since movement deviations were minimal or non-detectable. From the motion parameters, we calculated derivatives using the command,

1d_tool.py -infile “input.1D” -set_run_lengths 200 -set_tr 1.5 -derivative -overwrite -write “output.1D”.

To improve the signal-to-noise ratio and to reduce anatomical differences, we performed spatial smoothing by convoluting a Gaussian kernel filter with a size of 2.4 mm full-width-half-max (FWHM). The command,

*3dmerge −1blur_fwhm 2.4 -doall -prefix “output” “input”,* was used for in-volume spatial smoothing, and the output data was used for visualization. We created a brain mask using the command,

*3dAutomask -peels 5 -prefix “output.nii” “input.nii”,* allowing the removal of surrounding non-brain tissue and enabling the selection of brain voxels from the time series. Next, we calculated a mean *EPI* from the skull-stripping datasets for each run and calculated a grand average mean *EPI* for all the in-session data. We then used the mean *EPI* to calculate a non-linear warp between the mean *EPI* data and the in-session anatomy using the command,

*3dQwarp -source “input.anat.nii” -base “input mean.EPI.nii” -prefix “output.nii” -mi -verb -iwarp mi -blur 0 3 -Qfinal -verb -blur 0.*

The inverse transformation warp was then applied to the original time series using the command,

*3dNwarpApply -nwarp “WARPINV.nii” -source “input.nii” -master “input.nii” -prefix “output.nii”.*

After non-linear alignment, all data was then ready for the first pass regression and general linear modeling using *3dDeconvolve*.

### 2.7 General linear-modeling (GLM) analyses

For the first series of experiments, we presented two movie segments partitioned into five movie clips. Each segment lasted 30 s, followed by a 30 s period of no visual stimulation (darkness). We presented five movie segments (30 s each) within a run total of 5 min per scan (300 s, 1.5 s TR, n vol = 200).  For the input dataset to 3dDeconvolve(Step 9), we provided the detrended time-series and then applied ordinary least squares to a regression model of the BOLD signal, which included a gamma variate block design of the hemodynamic response to the movie period. We performed general linear T-tests between movie periods and the baseline/blank period to assess functional activation. Stimulus times were created with the script (*make_stim_times.py*) and included the volumes assigned to movie and baseline (e.g., 30 s ON/ TR = 1.5 s = 20 vol ones and 20 vol zeros). To visualize the block design, we used the commands (*3dDeconvolve* and *1dplot)*. General linear modeling was performed using (*3dDeconvolve*) and the output visualized within AFNI for evaluation of functional activation. The threshold was chosen as significant t-value (T-value colormap range 2.3 < 10) and at a corrected range for false discovery rate (FDR q value < 0.05). We confirmed significant activation across visual and higher-visual-related regions in all four macaque monkeys (**Supplementary Fig.2A**). GLM analyses were performed during data acquisition to evaluate the significance of BOLD response and to decide whether or not more data was needed. Overall, we observed a classical general pattern of activation in all four macaque monkeys, similarly to previous reports on NHPs visual fMRI experiments (Goense et al., 2012; Logothetis et al., 1999).

### 2.8 Diffusion data pre-processing

For the DWI data, we first corrected the volumes for slice-timing differences using the command,

*3dTshift -tzero 0 -prefix input.nii.*

We then averaged the five-volume repetitions on each dataset using the command,

*3dcalc -a input.nii'[0..63]' -b input.nii'[64..127]' -c input.nii'[128..191]' -d input.nii'[192..255]' input.nii'[256..319]' -expr '(a* *+* *b* *+* *c* *+* *d* *+* *e)/5′ -prefix “output.nii”.*

The average dataset was then aligned to a reference image using the command,

*3dAllineate -base “input.nii[0]' -input.nii -prefix output.nii -cost mi -verb -EPI*.

We then selected the 64 directional data using the command,

*3dcalc -a input.nii' [3..63]' -expr 'a' -prefix output.nii,* and entered the output into 3dDWItoDT (Step 5) to compute the six directional vectors (Dxx, Dxy, Dyy, Dxz, Dyz, and Dzz) and reference gradient vectors (Gxi, Gyi, and Gzi). With the command,

*3dDWItoDT -prefix output.nii -automask -reweight -verbose 100 -sep_dsets -eigs bvecs input.nii,* we obtained all vectors. Additionally, *3dDWItoDT* provided eigenvalues (L1, L2, and L3), eigenvectors (V1, V2, and V3), fractional anisotropy (FA), mean diffusivity (MD), and radial diffusivity (RD). We transformed the FA data into an RGB color-coded map using the command,

*3dThreetoRGB -prefix output.nii -anat input.nii'[0]'*.

The image shows the color-coded FA and overall white matter fiber bundles of each monkey (**Supplementary Fig.2B**). We then computed the uncertainty via jackknife of DTI estimates on-voxel-by-voxel basis using the command,

*3dDWUncert -inset input.nii -mask input.nii -prefix output.nii -input.tensors -grads bvecs -iters 50*.

To render the streamline tracts from the visual cortex we tracked white matter bundle pathways with the program 3dTrackID (Step 6). 3dTrackID is based on the fiber assessment by continuous tracking, including diagonals (Taylor and Saad, 2013). For diffusion tractography, we implemented the command,

*3dTrackID -mode MINIP -dti_in DTI.tensors -dti_extra mask.input.nii -netrois input.rois.nii -logic AND -mini_num 5 -uncert input.nii -alg_Thresh_FA 0.45 -prefix output.nii*

To track visual bundles, a pair of ROIS were selected of the same hemisphere from the atlas via their numerical index. We used the LGN as a source and V1 as a target ROI for tracking the optic radiation. To track the forceps major pathway, we selected V1 ROIS on both hemispheres, left V1 as a source and right V1 as a target (see **Supplementary Fig.6** and **7**).

### 2.9 Independent component analysis (ICA)

To reveal functional patterns during free-viewing we used independent component analysis (ICA) using the Multivariate Exploratory Linear Optimized Decomposition into Independent Components (MELODIC: http://fsl.fmrib.ox.ac.uk/fsl/fslwiki/MELODIC) of the FSL package. ICA estimates the consistency of a set of spatially and temporally overlapping components over the fMRI time series. Components might consist of meaningful organizing patterns such as those typically measured during resting state conditions in addition to other artifactual effects such as head motion, heart pulsation, or respiration, each carrying an independent spatial pattern and time course. We aimed at mapping the overall pattern during free-viewing from the spatiotemporally rich signals. The MELODIC ICA algorithm attempts to segregate the spatial overlap between the components based on the independence of the fMRI-BOLD signals. ICA is a “model-free” algorithm that aims to detect cortical and subcortical responses prevalent among a cluster of voxels, instead of the classical modeled BOLD response for comparing the fMRI signal. Previous NHPs studies suggested that the optimal number of components lies within the range of 20–30 independent components for RS-fMRI data (Mantini et al., 2012). Here we mainly aimed at replicating our previous results with GLM but with ICA. All runs across monkeys showed the predominance of the free-viewing pattern and the modulation of the BOLD signal by block design as the leading first component detected, explaining 7–14% of the variance (see **Supplementary Fig.3** and **Supplementary Table 2**).

### 2.10 Temporal signal-to-noise ratio

To demonstrate the quality of the awake macaque EPI data, we calculated the temporal signal-to-noise ratio (tSNR). The tSNR is the mean signal divided by the standard deviation of the signal over the voxel time series (Welvaert and Rosseel, 2013). We used the command,

*3dTstat -tsnr -prefix output.nii input.nii* to calculate the tSNR on the minimally preprocessed time series (e.g., after alignment to the anatomy). The temporal tSNR maps ranged between 0 and 100 for easy visual comparison across subjects (**Supplementary Fig. 4**). Additionally, we calculated the mean tSNR for each run acquisition which ranged between 46–82 across subjects and runs (see **Supplementary Table 2** for details). In general, the maps show high tSNR across gray matter structures, with more tSNR in temporal regions that were closer to the phase-array coil loops.

### 2.11 Coherence analyses

An additional useful model-free approach for mapping functional activation is the measured of coherence between the BOLD response to the stimulation frequency. Coherence measures the amplitude ratio at the fundamental stimulation frequency to the signal variance, ranging between 0 and 1 (Brewer et al., 2002). The measure of coherence is,

$$C\left(f_{0}\right)=A\left(f_{0}\right)/{\left(\sum_{f=\;f_{0}-\frac{\Delta f}{2}}^{f_{0}+\frac{\Delta f}{2}}A\;{\left(f\right)}^{2}\right)}^{\frac{1}{2}}$$

where, $f_{0}$ is the stimulus frequency,$\;A(f_{0})$ the amplitude of the signal at that frequency, A(f) the amplitude of the harmonic term at the voxel temporal frequency f and Δf the bandwidth of frequencies in cycles/scan around the fundamental frequency $f_{0}$. For all movie stimuli, $f_{0}$ corresponds to one cycle (1/60 s = 0.016 Hz), and Δf corresponds to the frequencies around the fundamental (see **Supplementary Fig. 6B**). We choose a threshold at a coherence level *>* 0.35. For all monkeys, the movie scenes within a single 5-minute-long scan (2 scan repetitions) elicited a significant BOLD response modulation (coherence *>* 0.35) in a large number of cortical areas, including occipital, temporal, parietal, and frontal regions (**Supplementary Fig. 6C** and **7A**).

### 2.12 Construction of free-viewing networks

In contrast to functional mapping analyses, network analyses enable the detection of regional hubs. Specialized regions that might become more relevant than others during the viewing of visual scenes. Towards this end, we construct free-viewing networks (see Fig. 1**C** and **Supplementary Fig. 1**) via the individualized brain parcellations of the D99 macaque brain atlas (Reveley et al., 2017). First, we obtained regional time series using the program (3dNetCorr). Next, we down-sampled the anatomical-based regions to match the underlying EPI 3D-grid (3dFractionize) and used each ROI to get the mean time series. The time series output from 3dNetCorr was then read into Matlab for connectivity measures. For movie one, the time-series were analyzed for both visual and dark periods.

Previous studies in humans have utilized the maximal overlap discrete wavelet transform (MODWT) for connectivity studies of resting-state fMRI and task-based conditions. Similarly, we used the MODWT to decompose each mean regional time series into wavelet scales corresponding to specific frequency bands (Sizemore and Bassett, 2018). The time-series were decomposed into wavelets using the orthogonal *Daubechies* wavelet function (https://uk.mathworks.com/help/wavelet/ref/modwt.html), which resulted in five-level decompositions ranging from 0.001 to 0.25 Hz. We concentrated on two levels (0.125–0.06) and (0.06–0.03), since hemodynamic events occurred within the movie viewing periods and at a relatively low-frequency range of 6 to 24 s. To demonstrate level decompositions, we constructed each matrix across the different wavelet levels (**Supplementary Fig. 5**). Overall, the network pattern remained across much of the level decompositions with the noise level increasing for higher frequency levels and decreasing for lower frequency levels. The correlation coefficients between the original matrix and levels 2 and 3 were highest, while lowest for high-frequency (level 5).

**Fig. 2:**
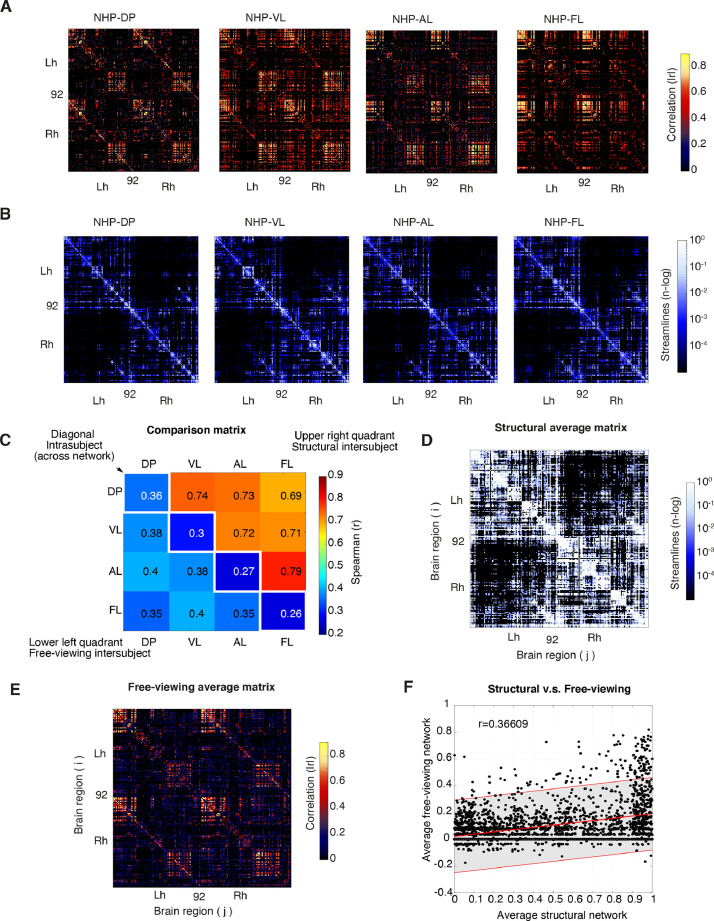
**Moderate relationship between free-viewing and structural networks. A**. Subject-specific, average free-viewing matrices, show the absolute correlation coefficient between every pair of mean time series from each ROI. **B**. Static structural matrices for each macaque monkey. **C**. Matrix showing the relationship between-subject structural (intersubject, upper right quadrant) and between-subject free-viewing networks (intersubject, lower left quadrant). The matrix shows the Spearman correlation coefficients (r) between pairs of matrices. Notice how the similarity and correlations among structural networks is relatively closer in comparison to free-viewing networks which showed more variance and relatively lower correlations. The diagonal highlights (white squares) the correlation within the same subject but across network types (e.g., within subject free-viewing networks versus structural networks or intrasubject). **D**. Average structural connectivity showing the logarithmic number of streamlines touching every pair of ROIs for both hemispheres. **E**. Average free-viewing matrix (184 × 184) matrix taken across subjects (*n* = 4). **F**. Scatter plot of correlation coefficients between average structural network and average free-viewing network with linear regression fit line (red) and 95% prediction interval (shaded gray area) showing a moderate positive correlation (Spearman's *r* = 0.37).

From the low frequency (0.125–0.03) fMRI signals, we then computed the temporal correlation between the activity of each pair of brain regions over the whole time series (e.g., average free-viewing networks). The weighted matrices (Fig. 2**A**) for each NHP subject were based on the absolute value in the pairwise correlations among regions. Importantly to consider when building functional networks is the potential of small non-zero values in the matrices, which may reflect the measurement of noise rather than the presence of an actual correlation (van Wijk et al., 2010). To overcome this potential issue, we applied a false discovery rate threshold (FDR p-value < 0.01) to the undirected adjacency matrices, $\left(A_{ij}\right)$ to determine which connections should be kept in the matrices.

$$\left(A_{ij}\right)=\;\{\begin{array}{c} C_{ij}\;if\;C_{ij}>t,\\ 0\;otherwise \end{array}$$

Using this threshold, the edges of $\left(C_{ij}\right)$ are a subset of the edges in $\left(A_{ij}\right)$. Subsequently, we mostly relied on the Brain Connectivity Toolbox (https://sites.google.com/site/bctnet/measures/list) to characterize network properties from the binarized matrices.

### 2.13 Construction of structural networks

The program 3dTrackID (Step 7) tracked DTI tensors along a pair of ROIs from the D99 atlas and provided the structural matrix. The output matrix from 3dTrackID is based on the number of streamlines touching each pair of ROIs. For creating structural networks, we detected the number of streamlines (*n >* 10) that connected any pair of regions *i* and *j*. We then arranged the logarithmically scaled number of streamlines on a 184 × 184 connectivity matrix (Aij) of brain regions (Fig. 2**B**) for each NHP subject. For characterizing the matrices and for subsequent network measures we used the binarized matrix.

### 2.14 Modularity of structural networks

We calculated network modularity (Q) via a hierarchical consensus algorithm. To calculate (Q) maximization, we compare the adjacency matrix $\left(A_{ij}\right)$ with the expected null connectivity model $\left(P_{ij}\right)$, where larger (Q) values indicate higher quality. The data-driven approach allows grouping nodes into modules that show high internal density as would maximally be expected from the null model. Consensus modularity (Q)is defined as:

$$Q\left(y,\left\{g_{i}\right\}\right)=\sum_{ij}\left[A_{ij}-\gamma P_{ij}\right]\left[\delta_{\left(gi,gj\right)}\right]$$

where γ is the resolution parameter used for optimization; $g_{i}\epsilon \left\{1,\ldots ,C\right\}$ is the module assignment of node i, where the Kronecker delta function $\delta_{\left(g_{i},g_{j}\right)}$ equals one, if nodes i and j belong to the same module ($g_{i}=g_{j}$). The function ensures the total weight of within-module edges is less than that of the null model. We used the multiresolution consensus function to calculate modularity (Jeub et al., 2018) (https://github.com/LJeub/HierarchicalConsensus).

### 2.15 Centrality in free-viewing networks

To find the most critical–or central–regions in the network, we calculated eigenvector centrality (EC) for each brain region (Lohmann et al., 2010). Eigenvector centrality (EC) measures the degree of a node's influence in the network by scoring the node's eigenvalue. A high eigen value indicates that the node is well-connected to many other well-connected nodes. For the threshold matrix $\left(C_{ij}\right)$, let $x_{i}$ be the eigenvector centrality of node i and λ largest eigenvalue and x the corresponding eigenvector. Eigenvector centrality is defined as,

$$x_{i}=\frac{1}{\lambda }\sum_{j=1}^{n}C_{ij}x_{j}$$

The proportional factor $\frac{1}{\lambda }$, is such that $x_{i}$ is proportional to the sum of similarity scores of all connected nodes. We used the Brain Connectivity Toolbox to calculate eigenvector centrality. The MATLAB function is available at the Brain Connectivity Toolbox website (https://sites.google.com/site/bctnet/measures/list).

### 2.16 Time-varying functional connectivity of free-viewing networks

Time-varying functional connectivity was estimated using the multiplication of temporal derivatives (MTD), which calculates each sample product of temporal derivatives for pairwise time series (Shine et al., 2015). For each time point t, the MTD is defined for the pairwise interaction between region i and j,

$$MTS_{ijt}=\frac{1}{w}\sum_{t}^{t+w}\frac{\left(dt_{it}\times dt_{jt}\right)}{\left(\sigma_{dt_{i}}\times \sigma_{dt_{j}}\right)}$$

Where dt is the first temporal derivative of either i and j at time t, and σ is the standard deviation of the temporal derivative for time series i and j, and w is the window length (n samples = 12 × 1.5 TR = 18 s) of the moving average. For epoch specific periods, we average over matrices points of the presentation period. See coupling for more details (https://github.com/macshine/coupling/).

## 3 Results

In the present study, we characterize the macaque functional brain connectivity and its relationship with the underlying neuroanatomy. From the structural connectivity, we evaluate the static architecture of each NHP network, while from the functional connectivity we aim at first assessing the average functional connectivity (Experiment movie 1) and then the dynamic functional connectivity (Experiment movie 2, see Fig. 1). Prior to the implementation of time-varying functional connectivity, we characterized the properties for each NHP subject network within and across hemispheres.

### 3.1 Moderate relationship between free-viewing networks and structural neuroanatomy

The connectivity matrix forms the basis of all network measures and is essential to quantify its properties prior to additional measures. To quantify free-viewing matrices of each NHP (Fig. 2**A**), we calculated network density (ρ), which is proportional and varies between zero and one, where ρ=0indicates no connection available, while ρ=1 indicates that all possible connections exist, and 0<ρ<1 represents the fraction of all possible connections that are present in the network. We summarize all density measures for each monkey and for each hemisphere of both free-viewing and structural networks in **Supplementary Table 3**.

For free-viewing matrices, the density from half of the matrix corresponding to the left hemisphere was similarly dense as that of right hemispheres (Lh, μ = 0.16, ± σ = 0.01; Rh, μ = 0.21, ± σ, = 0.05). The density over the whole brain (μ = 0.17; σ = 0.03) did not differ largely from the connectivity density of a single hemisphere alone, indicating the existence of similar functional properties in both left and right hemispheres. Such symmetrical features in the network could also be appreciated from visual inspection of the free-viewing connectivity matrices of each NHPs subject.

In contrast, the density of structural networks showed higher intrahemispheric density (ρ) (Lh, μ = 0.42, ± σ = 0.03; Rh, μ = 0.40, ± σ = 0.04) as compared to the total density when sampling both hemispheres (both; μ = 0.28, ± σ = 0.02). Such smaller connection density across hemispheres (interhemispheric) than within the hemisphere (intrahemispheric) largely reflected the smaller number of large distance streamlines passing through the corpus callosum. The smaller interhemispheric connections in structural networks could also be appreciated from visual inspection of the matrices of each NHP subject (Fig. 2**B**). Similar results were described in human anatomical brain networks derived from DTI (Bonilha et al., 2015).

To better estimate the similarities or differences among free-viewing and structural networks, we calculated the Spearman correlation coefficient (r) between any pair of matrices. The intersubject correlation among structural networks showed high correlations coefficients (see upper right quadrant of the matrix in Fig. 2**C**, range of correlation coefficients (r) = 0.60–0.79) indicating an overall similarity shared across NHPs structural networks. In contrast, intersubject correlations of free-viewing networks showed relatively lower correlations (see lower left quadrant of the matrix in Fig. 2**C**, range of correlation coefficients (r) = 0.35–0.4) than structural networks, suggesting an increased variability among free-viewing networks. To evaluate intra-subject correlations–the correlations between free-viewing and structural networks of the same subject–we also calculated the Spearman correlation coefficient (r) between matched pairs of structural and functional networks. These comparisons showed relatively low correlations (see diagonal of the matrix in Fig. 2**C**, range of correlation coefficients, (r) = 0.26–0.36) between structural and functional networks of the same subject indicating stark differences between networks.

To further evaluate the differences between networks, we extended our analyses by evaluating the relationship between function and structure per node. Towards this end, we averaged all NHP subject structural matrices into a grand average structural matrix (Fig. 2**D**) and performed the same average for free-viewing matrices (Fig. 2**E**). From the two averaged matrices we then computed the correlations per node and fitted a linear regression line (see Fig. 2**F**, Spearman's *r* = 0.37, linear fit 95% prediction interval) which at most indicated a moderate positive relationship, indicating an imperfect correspondence between structure and function in NHP macroscale networks.

In summary, structural networks show differential connectivity architecture as compared to free-viewing networks. We demonstrated a) a higher interhemispheric density in free-viewing networks as compared to structural networks, b) denser intrahemispheric connections in structural than free-viewing networks, c) closer similarity among subjects’ structural networks, d) greater variability in subjects free-viewing networks and, e) a moderate positive correlation between free-viewing and structural networks.

### 3.2 A closer relationship between free-viewing and structure in visual subnetworks

**Fig. 3:**
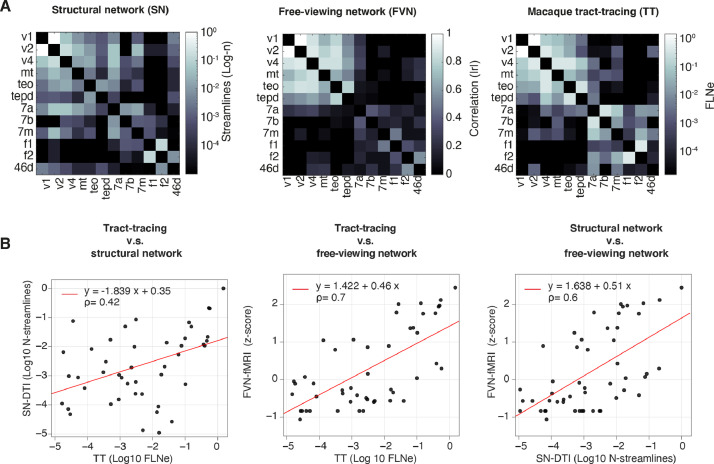
**Closer relationship between free-viewing and structure in visual subnetworks A**. Partial network matrices: structural matrix (based on DTI, left), free-viewing matrix (based on fMRI, center), and macaque anatomical matrix (based on track tracing, right). **B**. (Left) scatter plot of vectorized Log transformed tract-tracing, and structural-DTI matrices. The legend shows estimated regression values along with rho (*p*) values with the linear fit regression line shown in red. (Center) semi-log plot for tract-tracing and free-viewing (z-scored) and (right) structural-DTI versus free-viewing. Notice a higher correlation between free-viewing and track-tracing or with DTI-based structural subnetworks.

To further expand the relationship between free-viewing and structural networks, we then compared all identified networks with those obtained from available tract-tracing macaque data. Toward this end, we matched the ROI label nomenclature of the regions from the F99 atlas (Markov et al., 2014), to the D99 (Reveley et al., 2017). We found twelve matching ROIs and used these ROIs to compare networks across modalities. The average partial matrices (Fig. 3**A**, see **Supplementary Fig.13** for each partial monkey matrix) were used to evaluate the relationship between networks across modalities. The Rho value correlation between free-viewing and structural track-tracing networks showed relatively high rho values (*p* = 0.7) and similarly to the DTI-based structural networks (*p* = 0.6, see Fig. 3**B**). In contrast, tract-tracing versus DTI-based networks show good correspondence but lower rho values (*p* = 0.42).

The higher correspondence between macaque structural and free-viewing visual subnetworks highlighted a closer relationship between structure and function, largely reflecting an increase in functional connectivity during visual processing of the movie scenes.

### 3.3 Modularity and centrality reveal hubs within frontoparietal lobules forming a visuo-saccadic network

#### 3.3.1 Node degree of structural and free-viewing networks

**Fig. 4:**
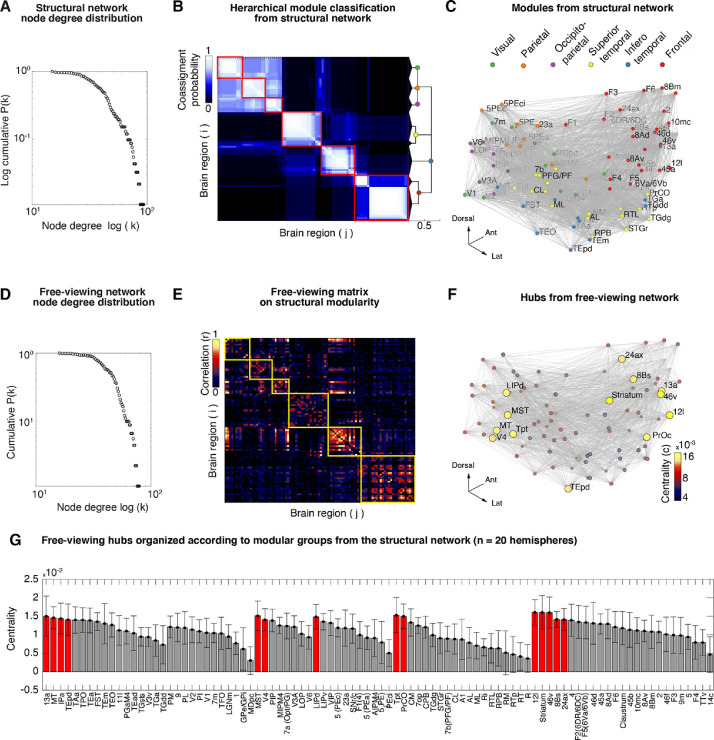
**Centrality and modularity reveal hubs forming a visuo-saccadic network in occipito-parietal and frontal modules A**. Complementary cumulative distribution function (cCDF) of node degree of structural network shows a decrease in the probability of finding a highly connected done (*k*), indicating the existence of a small number of nodes with high connections. **B**. Co-assignment matrix obtained from the average structural connectivity (*n* = 8 hemispheres) and hierarchical dendrogram highlights module classification (red squares) based on a data-driven Q-value maximization algorithm. **C**. Modules plotted in 3-dimensional space with nodes (visual, occipitotemporal, temporal, occipitoparietal, parietal, and frontal modules) color-coded according to their modular assignment. **D**. The complementary cumulative distribution function (cCDF) of node degree of free-viewing network shows a decrease in the probability of finding a highly connected node (*k*), similarly indicating the existence of a small number of nodes with high connections. **E**. Average free-viewing network (92 × 92) matrix from each subject and hemisphere (*n* = 8) and organized according to the structural modularity obtained from the structural network above. **F**. Hubs with high eigenvector centrality which measures the degree of a node influence in the network by scoring the node's eigenvalue. A high-eigenvalue indicates that a node is well-connected to many other well-connected nodes. For the average free-viewing networks we chose to highlight the topmost central regions with an eigenvector centrality value (EC) *>* threshold (t) = 14 × 10^−3^ corresponding to a z-score *>* 1. The 3-dimensional graph highlights hub regions that formed a visuo-saccadic network. **G**. Rank centrality averaged (mean +/- std) over 14 hemispheres shows the consistency in hubs during free-viewing. Groups are shown for the static modular architecture obtained from structural networks. **Supplementary Fig. 15** shows the centrality networks for each monkey hemisphere.

A basic principle in network science is understanding how connectivity varies between nodes. A simple calculation is to count the number of connections each node has with respect to the rest of the network, a measure called node degree. Node degree (κ) measures the number of edges (e.g., number of streamlines in structural networks) of each node (i) with all other nodes (j) in the network. In most biological networks, the distribution of node degree is heterogeneous, with a large number of nodes sharing a few connections while a smaller number of nodes take a large number of connections, making them putative hubs since the small number of nodes have the potential to integrate information in the network. We evaluated the cumulative distribution function (CDF) of node degree (κ) in structural networks which showed a decay in the probability of finding a node with high node degree (see Fig. 4**A** for average network and **Supplementary Fig. 10** for each independent hemisphere). The (κ) range across macaque structural networks (κ) was 148–159. For free-viewing networks, node degree (κ) ranged between 58–86 across subjects indicating the existence of a small number of nodes with a large number of connections (e.g., ∼70 *>* connections, see Fig. 4**D**, for each monkey and hemisphere, see **Supplementary Fig. 8**).

Node degree of both structural and free-viewing networks showed a distribution decay indicating the existence of a large number of nodes with few connections and a smaller number of nodes with many connections. The small number of nodes with many connections are considered essential in brain networks, as their increased connectivity enables global and efficient communication (Bassett and Sporns, 2017).

#### 3.3.2 Clustering coefficient of structural and free-viewing networks

Anatomical tract-tracing (Markov et al., 2014) and graph-theoretical studies (Song et al., 2014) established that brain regions in macroscale networks tend to be densely connected with proximal neighboring regions. By measuring the clustering coefficient (C), we could quantify the number of connections between the node's nearest neighbor as compared to the total number of all possible connections. To evaluate the validity of clustering coefficients, we require random networks which tend to have on average lower clustering than normal brain networks (Bullmore and Sporns, 2009). C takes a value between zero and one, where a value of zero indicates lack of clustering while a value of one indicates a fully connected network.

For the binarized structural networks, C showed a relatively higher value (see **Supplementary Fig. 12A** and **Supplementary Table 3**, NHP range: 0.69–0.71; μ = 0.7, ± σ = 0.01) as compared to rewired networks with the same degree distribution under the null- hypothesis (NHP range: 0.45–0.52, μ = 0.5, ± σ = 0.69 × 10^−3^). For the binarized free-viewing networks, C showed similarly higher values (see **Supplementary Fig. 12C** and **Supplementary Table 3**, NHP range: 0.48–0.62; μ = 0.5, ± σ = 0.001) as compared to rewired networks with the same degree distribution under the null-hypothesis (NHP range: 0.12–0.24, μ = 0.18, ± σ = 0.001).

The clustering coefficient analyses indicated that both structural and functional networks show the topological capacity for integration in local subgraphs with the potential to facilitate functional specialization (e.g., segregation) known to be critical for efficient network communication (Hilgetag and Kaiser, 2004; Kaiser and Hilgetag, 2006).

#### 3.3.3 Modularity of structural networks

Network modules are groups of densely connected regions that are sparsely connected across groups (Sporns and Betzel, 2016). To identify network modules from the average structural matrix, we used modularity maximization, a data-driven approach for the hierarchical clustering of nodes into network groups or modules (Betzel, 2020). Using this approach, we found six hierarchical partitions that captured lobular brain organization (Fig.4**B** and **Supplementary Fig. 16A**). The organized brain structure is evident on the 3-dimensional anatomical network plot, where regions preserve their center of mass and anatomical coordinate. The nodes formed visual, occipitotemporal, temporal, occipitoparietal, parietal, and frontal groups (Fig.4**C**). Some of the modules showed the ability to further subdivide within the hierarchy (e.g., frontal module). However, the six levels subdivisions largely recapitulated the macaque overall structural brain connectivity (Harriger et al., 2012). To ease the visualization of the connectivity structure, we illustrate the network in a circular dendrogram with hierarchical bundling of the edges to reduce visual clutter (Holten, 2006). The bundle edge technique allowed us to map the implicit adjacency across modules and connectivity within regions (see **Supplementary Fig. 16B**). Additionally, on the same graph, we mapped the number of connecting edges to each node or node degree (κ) on the node face size. The visualization technique allows mapping the most important network features into a single graph enabling a complete summary of the macroscale connectivity architecture.

Modularity of structural networks largely captured the well-known anatomical organization of the macaque brain (Harriger et al., 2012) using a data driven approach. Furthermore, our findings also demonstrate the potential of structural networks derived from DTI in awake monkeys for mapping the macroscale organization of the macaque anatomical brain network (Hilgetag and Kaiser, 2004; Kaiser and Hilgetag, 2006; Markov et al., 2014). Most importantly, the organized modules allowed us to obtain the static architecture critical for inferring changes in functional brain networks.

#### 3.3.4 Centrality of free-viewing networks

Very often, functional activation reveals regions with different degrees of activation strength challenging the inference and extent to which a region engages in a particular cognitive task. Network centrality instead allows us to identify brain regions with high importance by measuring the degree of a node's influence in the network and by scoring the node's eigenvalue (Bassett and Sporns, 2017).

In brief, a high eigenvalue indicates that a node is well-connected to many other well-connected nodes. For the average free-viewing networks, we chose to highlight highly central regions with an eigenvector centrality value (EC) threshold (t) *>* 14 × 10^−3^ corresponding to a Z-score *>* 1. Using this method, we identified a large-scale network engaged during movie watching (Z-score *>* 1 average free-viewing network; EC = 14 × 10^−4^) with the following functional hubs: MT/MST, V4, TEpd, IPa, Tpt, LIP, 8B, 46v, ProC, 12l, 13a, and striatum (Fig. 4**F**). Many of the regions are known to carry out specific visual functions related to object recognition, visual motion processing, eye movement control, and visual attention (Buschman and Kastner, 2015; Gilbert and Li, 2013; Russ and Leopold, 2015; Russ et al., 2016). The centrality for each independent NHP subject showed variable patterns of centrality (see **Supplementary Fig. 15**), however hub regions were mostly observed within frontal and occipito-parietal modules. The average centrality taken over each monkey, runs and hemispheres (*n* = 20) showed consistent patterns of centrality highlighting a visuo-saccadic network (Fig. 4**G**).

While we observed many cortical regions known to have roles in visual function (e.g., LIP, FEF, MT/MST, V4, TEpd, IPa), the centrality measures enabled the identification of additional hubs that included (Tpt, 46v, ProC, 12l, 13a, striatum). Many of these additional regions are not well delineated in terms of their contribution to visual function. However, half of the identified regions are known to belong to a group of ‘rich clubs’ of areas or hubs (e.g., TEpd, LIP, 8B, 46v, 12l, and 13a) as derived from structural connectivity analyses of the macaque monkey (Harriger et al., 2012). These regions in the macaque connectome are thought to be part of a network that could facilitate global network communication.

Centrality during free-viewing revealed a widespread long-range network of cortical and subcortical hub regions consistently present in each monkey. To explore the state of the network further we measured the edge distance of the long-range interactions present during free-viewing.

### 3.4 Path length and long-range interactions during free-viewing

#### 3.4.1 Path length of structural and free-viewing networks

Previous network studies in both humans and monkeys indicated that multiple network properties emerged from the anatomical location and distance of regions within the brain (Hilgetag and Kaiser, 2004). An increase in local connectivity results in networks that prefer short-range connections, reflecting intrinsic structural properties of brain connectivity that reduce the metabolic cost of synchronizing activity among a wide range of brain areas. The distance between local or clustered nodes tend to be short, thus anatomically nearby regions are therefore considered to be “economical”, whereas the edges of long-range projections are fewer and sparse but had the capacity for integration over the network (Watts and Strogatz, 1998).

**Fig. 5:**
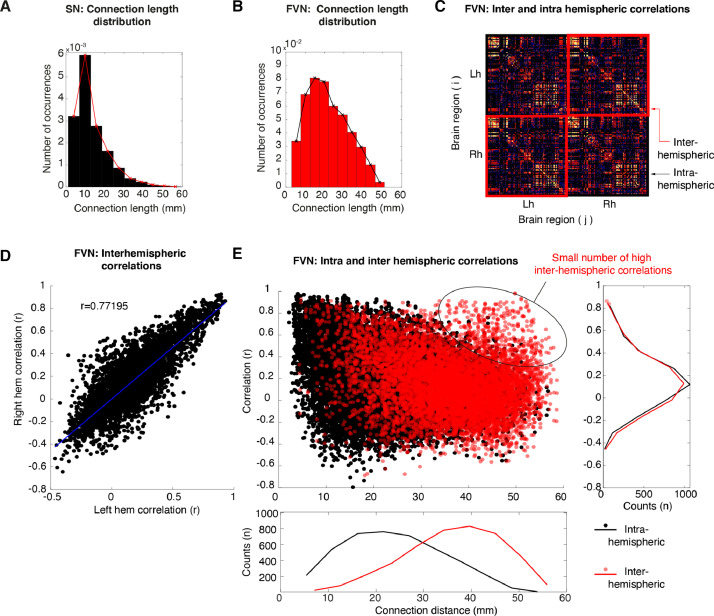
**Highly-weighted long-range connections across the cerebral hemispheres indicate large-scale integrative properties during free-viewing. A**. Distribution of path lengths shows a sharp decay as a function of Euclidean distance for structural networks. **B**. In contrast, the distribution of path lengths for free-viewing networks shows a wider monotonic decay as a function of Euclidean distance. Such increases in large-scale connections might enable a neuronal network to integrative information across widespread interareal interactions. **C**. Connectivity matrix organized from left to right hemispheres. **C**. Scatter plot of left and right hemisphere correlations showing high similarity across hemispheres. **D**. Scatter plot of node distance and correlation magnitude showing inter (black) and intrahemispheric correlations (red). The outset panel to the right shows the average counts of correlation coefficients. The outset bottom panel shows the average counts of distance lengths. Both correlations decrease as a function of distance. However, a small number of long-distance interhemispheric correlations remains.

The characteristic path length (L), measures the average shortest path for all possible pairs of nodes in the network or the average number of steps to reach from one node to another. We explored the characteristic path length (L) of binarize structural matrices which showed a classical shorter path length (L range across NHPs, structural networks: 1.73–1.8 mm, μ = 1.76 mm, ± σ = 0.001) as compared to the null-model rewired networks (see **Supplementary Fig. 12B**, L rewired structural networks: 1.7–1.75 mm, μ = 1.72 mm, ± σ = 0.4 × 10^−5^). The connection length distribution (Fig. 5**A)**, showed a peak distance between 10-12 mm and a exponential decay as a function of distance consistent with previous reports of anatomical tract-tracing studies in the macaque (Markov et al., 2014). In contrast, the average path length of free-viewing matrices was relatively longer as compared to structural matrices (μ = 2.21 mm, ± σ = 0.04, range: 2–2.5 mm,) and showed greater differential distance as compare to their respective null-model rewired matrices (see **Supplementary Fig. 12D,** L rewired μ = 1.8 mm, ± σ = 0.002, free-viewing networks: 1.8–1.9 mm). **Supplementary Table 3**, and **Supplementary Fig. 11** summarizes the characteristic path length for each hemisphere and macaque network.

Important to clarify is that the measure of path length in correlation-based functional networks is susceptible to interpretation challenges, given that a strong correlation does not necessarily indicate the presence of physical anatomical connection. Furthermore, strongly correlated activity might result from one or more indirect, multi-synaptic connections (Rubinov and Sporns, 2010). Importantly to consider, is the fact that path length in functional networks represent sequences of statistical associations, and might not necessarily represent an actual route of information flow. Despite their interpretational difficulties, short-paths in functional networks could potentially capture direct connection via suppression of indirect connections (Marrelec et al., 2009). To further explore connection lengths in free-viewing networks, we measured the connection weights (e.g., correlations coefficients) as a function of their Euclidean node to node distance (node center of mass). We found that the degree of correlation decreased monotonically as a function of node distance (Fig. 5**B**), but with a peak distance around 15–20 mm (see **Supplementary Fig.8** for each monkey and hemisphere). Such widespread high-weighted correlations could reflect integrative properties during cognitive processing in free-viewing. To further explore this feature of free-viewing networks, we analyzed correlations across hemispheres (interhemispheric) and within hemispheres (intrahemispheric, see Fig. 5**C**). The interhemispheric relationship showed a positive correlation (Spearman's *r* = 0.77, Fig. 5**D**) indicating similar patterns of connectivity within each hemisphere (also evident from visual inspection of the connectivity matrix in Fig. 5**C**). Furthermore, we dissociate between interhemispheric and intrahemispheric correlations and show that the majority of the highly-weighted interhemispheric weights connections were found beyond 20 mm with a peak at 40 mm (Fig. 5**E**). These findings demonstrate the existence of long-distance associations with anatomically distal related regions across the cerebral hemispheres presented during free-viewing.

In sumarry, edges of both structural and free-viewing networks showed a monotonic decrease in path length. The path length distribution of structural networks was consistent with the interareal distances previously described for the cerebral cortex of multiple mammalian species (Song et al., 2014). Interestingly, for free-viewing networks we found a small number of long-distance interhemispheric correlations that might serve as additional functional architecture for the synchronization of regional activity over large distances during cognitive processing (Stisoand Bassett, 2018).

#### 3.4.2 Time varying functional connectivity during free-viewing

Previous analyses highlighted the existence of highly-weighted long-range correlations that we hypothesized had the potential to re-configure depending on the categorical changes coming from the stream of visual scenes. Furthermore, from the structural analyses we capture the static architecture (see **Supplementary Fig. 16B**) of each macaque brain network. Using this fixed architecture as underlying template, we then aimed to characterize any change in hub centrality and interareal connectivity during free-viewing that departed from the overall modular classification.

**Fig. 6:**
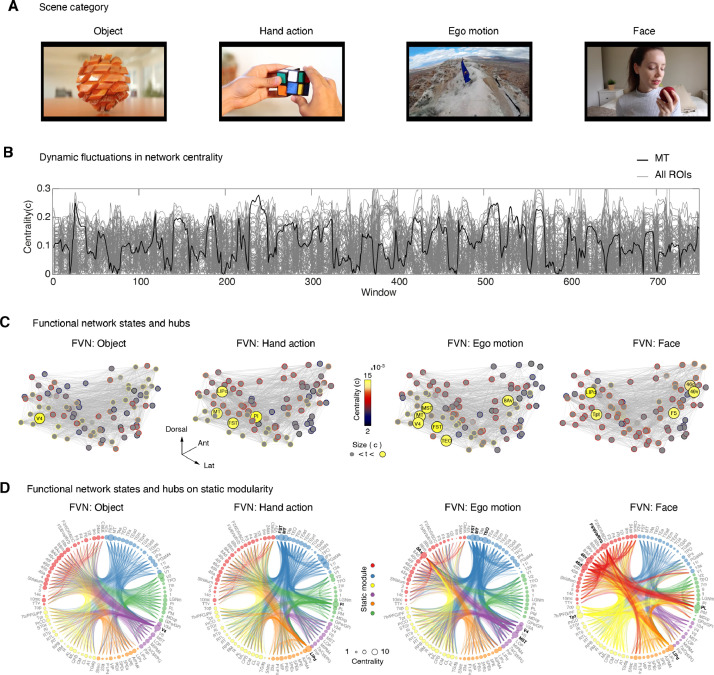
**Dynamic reconfiguration of free-viewing network hubs. A**. Example categorical image from the scenes presented to the NHPs during natural free-viewing of movie sequence two. The presentation periods (30 s each) include object, hand action, ego-motion, and face scenes. **B**. Dynamic fluctuations in network centrality. Gray plots show all ROI's centrality as a function of the sliding window. Black trace shows an example region (MT) with dynamic fluctuations in centrality during free-viewing. **C**. Eigenvector centrality (c) identifies network hubs (*c >* 1_×_10^−4^; n for size <= 6), highlighting the central nodes during the presentation of each main categorical scene. The hubs change across the different scenes highlighting differential network states during natural free-viewing. **D**. Hierarchical edge bundling allows visualization of connectivity patterns in free-viewing networks for each scene category on the underlying structural network modularity (face node color code). The node size illustrates the overall network centrality value for all nodes with the threshold indicated on the black color of the node label. The edges that touch a highly central region show an increase in the edge thickness (size = 1).

Since several regions showed specific scene-content-dependent activation (**Supplementary Fig. 14**), our next aim was to understand how functional interactions between areas might change across the different movie epochs (Fig. 6**A**). To this end, we built on our previous analyses and calculated network centrality for all regions during a moving time window.

The overall network showed dynamic changes in network centrality throughout the entire MRI scanning period (see example fluctuations in centrality from area MT in Fig. 6**B**). Interestingly, while the overall global centrality pattern largely reflected the induced sensory modulation, we observed that many regions significantly vary in their centrality across both the movie and darkness periods. From the specific scene epoch matrix, we calculated eigenvector centrality (Z-score *>* 1, *c >* 1 × 10^−4^ for n for size < = 6) and identified network hubs for each main scene category (Fig. 6**C**).

As a first instance, we observed changes in network centrality across all four original movie scenes. To ease visualization of regional interactions for each scene, we organized the network in a circular dendrogram with the node face size representing degree centrality. We used the modular classification of the static structure to classify each region. The edge-width highlights the principal projection bundles between hubs in the network, while the widths of all other edges were kept constant (Fig. 6**D**). In what follows, we describe the functional connections for each network state (e.g., object, hand action, ego-motion, and face) and relate connectivity patterns to existing knowledge about macaque anatomical connectivity of the hub regions identified using centrality.

For the object scene, we observed high network centrality in area V4 (see the occipitotemporal module color-coded in purple). The majority of connections to or from V4 diffuse into prefrontal (red) and superior temporal (yellow) modules. The high network centrality of V4 and its connectivity to prefrontal and temporal lobe areas observed during the object scene reaffirms the importance of this connectivity for object recognition (Roe et al., 2012). The interactions we observed between V4 and 8B (FEF) were also consistent with the reciprocal anatomical connections between both areas (Markov et al., 2014) and the proposed importance of this network in visuo-saccadic planning and attention (Roe et al., 2012).

Dynamic changes in centrality occur from passively viewing the moving object scene (in allocentric perspective) towards the more actively manipulated ruby cube scene (in egocentric perspective). This scene change re-configured the functional network. For the hand-action scene, we found high centrality in regions MT, FST, LIPd, and subcortically in the inferior pulvinar (PI) nucleus. Regions MT, FST show interactions that associate all five network modules with convergence in areas LIP in the parietal module (orange) and the PI nucleus (visual module in green). Area LIP is known for his role in visuospatial attention (Buschman and Kastner, 2015) and for the spatial coordination of limb movements (Oristaglio et al., 2006), which in accordance with the connectivity patterns we found for the hand-action scene. Further, we identified functional connections between the inferior pulvinar and area MT/MST, an anatomical connection previously described from tract-tracing studies in the macaque (Boussaoud et al., 1990; Kaas and Lyon, 2007). Moreover, dorsal motion areas are also known to be anatomically connected with the posterior parietal cortex, including area LIP (Boussaoud et al., 1990; Markov et al., 2014), supporting our network results. For comparison, during the hand-action scene, the V4 centrality observed during the object viewing vanished and its hub centrality became dormant.

A similar network configuration occurred again during ego-motion viewing. Though V4 was again involved in the processing of this scene, the overall recruited network showed stark differences in comparison to the object scene pattern, which recruited additional hubs in MT, FST, TEO, with functional connections between these hub regions and pre/frontal lobe region such areas 8av (e.g., FEF region). Anatomically, it is well established that areas V4, TEO, MT, FST, MST are inter-connected, linking information processing between dorsal and ventral visual pathways for motion analyses (Boussaoud et al., 1990). Our findings with the ego-motion scenes recapitulate recent fMRI studies in macaques, indicating a prominent visual motion drive during free-viewing of visual scenes (Russ and Leopold, 2015; Russ et al., 2016).

The macroscale network configuration dynamically adjusted once more with the onset of scenes that contained faces. For the face network, we found hubs in areas LIP, Tpt, 46, ventral premotor cortex (e.g., areas F5/6Va/6Vb), and subcortically the lateral pulvinar nucleus (PL). These results indicated that our centrality measures could capture additional features of the interareal interactions not available with standard GLM contrast analyses. While area V4 is sometimes reported in traditional face activation studies (Tsao et al., 2003), it does not appear to be part of the face processing network we captured. More specifically, interareal interactions for the face scene within the frontal module showed increases in local connectivity and with large edges connecting regions in the superior temporal module (area Tpt), parietal (area LIP), and visual (lateral pulvinar) modules. The lateral pulvinar is known to anatomically project to the ventral cortical stream (Kaas and Lyon, 2007) and to receive projections specifically from cortical face patches in the temporal lobe (Grimaldi et al., 2016). Moreover, the ventrolateral and ventral premotor cortex is connected to regions in the superior temporal and parietal cortex (Petrides and Pandya, 2009), a pathway that integrates social facial gestures (Shepherd and Freiwald, 2018) with vocal sounds (Ortiz-Rios et al., 2015). Our centrality measures further implicate this network in social communication (Sliwa and Freiwald, 2017) as similarly found in human brain networks during movie viewing (Lahnakoski et al., 2012).

In summary, connectivity analyses allowed us to observe changes in network interactions across cortico-cortical and thalamocortical network pathways. The dynamic changes we observed highlight the importance of the ongoing motor and cognitive interactions present during more naturalistic experimental conditions.

## 4 Discussion

In this study we used a free-viewing paradigm combined with graph-theoretical analyses to investigate how functional networks reorganize during active natural vision. Importantly, varying functional connectivity revealed specific thalamocortical and cortico-cortical functional interactions that reconfigured depending on the passive viewing of visual objects, motion, faces, and actions present within the scenes. Overall, across monkeys and movie scenes, we found a consistent functional network engaged during free-viewing that included hub regions in frontal (FEF), parietal (LIP, Tpt), and occipitotemporal cortex (MT, V4, and TEpd), among others.

In the following section, we discuss our results concerning the current knowledge of the anatomical connectivity between hub regions and the involvement of these regions in visual cognitive processes. Lastly, we envision how new naturalistic paradigms, combined with network neuroscience, can help us gain new insights into the dynamics of macroscale networks in NHPs.

### 4.1 Network state configurations during natural vision

One fundamental principle of brain organization is hubs in networks, which enable efficient neuronal communication and the integration of information over long distances (van den Heuvel and Sporns, 2013). By applying graph-theoretical methods, we were able to identify regions beyond the traditional visually engaged areas. These regions included multimodal areas (Tpt, 46v), the prefrontal cortex (12l, 13a, ProC), pulvinar, and the striatum.

When we focused analyses on movie scenes that contained faces, we found hubs in parietal (LIP), superior temporal (Tpt), prefrontal (area 46), premotor (F5), and subcortically in the lateral pulvinar nucleus. The dorsal network that connects premotor regions with the superior temporal and parietal areas (Petrides and Pandya, 2009) is known to integrate social facial gestures (Shepherd and Freiwald, 2018) and vocal sounds (Ortiz-Rios et al., 2015). The hubs we found in the frontal and premotor cortex (e.g., 46 and F5) along with the superior temporal (Tpt) region suggest that the free-viewing of faces engaged this network for the extraction of higher-order social features within the scenes (Shepherd and Freiwald, 2018). The projections from the superior temporal region (Tpt) and parietal cortex (LIP) may provide multimodal and spatial information to the face-sensitive areas in the IT cortex (Cusick et al., 1995; Seltzer and Pandya, 1994). Subcortically, we also found high centrality of the lateral pulvinar (PL) during the face scene periods. The PL anatomically projects to the inferotemporal cortex (Kaas and Lyon, 2007) and receives projections specifically from face patches (Grimaldi et al., 2016). An additional interesting point relates to the medial pulvinar (ML), which projects to the anterior STG and functionally is involved in higher-level features of vocal sounds. Both thalamic nuclei (medial and lateral pulvinar) might be a source for the modulation of vocal and facial information at the STS level (Bruce et al., 1981; Grimaldi et al., 2016; Scott et al., 2017; Smiley and Falchier, 2009).

Our analysis also revealed the recruitment of a different network engaged in the processing of hand action scenes. For these, we observed specific connections with areas LIP, MT/MST, and the inferior pulvinar (PI). Regions in the dorsal stream (e.g., LIP, MT/MST) are well-known to coordinate visuospatial information from eye and limb movements (Oristaglio et al., 2006). Moreover, these areas receive projections from the inferior pulvinar (Boussaoud et al., 1990; Kaas and Lyon, 2007), consistent with the observed connectivity patterns for the hand-action network. In the cortex, we found regions FEF, LIP, 46, 13, 24, F5, and TE as network hubs, in line with previous graph-theoretical analyses of macaque anatomical networks (Harriger et al., 2012). Area TE corresponds to the well-known AF face patch region of the inferotemporal cortex (Tsao et al., 2003) and their neurons respond to the spatial layout of natural visual scenes (McMahon et al., 2015). Additionally, area TE response to complex biological actions and received projections from the overlying poly-sensory STS region (Bruce et al., 1981) and parietal cortices, which might provide TE regions with multimodal spatial information.

Additionally, we also found recurrent network hubs, such as the motion complex network (e.g., areas MT/MST/FST), that emerge as central during the presentation of multiple visual scenes. Not surprisingly, the emergence of the motion complex network during free-viewing indicates the necessity for processing a substantial increase in visual complexity and visual motion in our selection of visual scenes. These findings also follow recent macaque fMRI studies using naturalistic stimulation that investigated the influence of self-induced and visual motion (Russ et al., 2016) and found a strong dominance of visual motion in the fMRI activation patterns along the STS.

Similar to MT hubness, we also found high centrality in area V4, which is known to be involved in visual shape and object recognition (Roe et al., 2012). For our object scene condition, it follows the role of area V4 in processing object features. The object network showed stark differences in comparison to the ego-motion network. The ego-motion network engages beyond V4 and incorporates motion complex regions (MT/MST FST). Additionally, area V4 plays a role in visuospatial attention (Moran and Desimone, 1985; Roe et al., 2012) and receives inputs from and projects to the FEF region (Markov et al., 2014) following our findings showing high centrality for area FEF. The connections we observed in free-viewing networks confirm the role that area FEF and V4 play in top-down visuo-saccadic functions (Ekstrom et al., 2009; Moore and Armstrong, 2003; Roe et al., 2012). Additionally, object motion-related regions (e.g., V4, MT, MST) became functional hubs along with saccade-related areas (e.g., LIP and FEF), indicating that, in general, movie viewing strongly engages this network configuration. Moreover, these results are in close accordance with previous ego-motion fMRI studies in macaques (Cottereau et al., 2017), showing the involvement of MST and FEF during ego-flow motion conditions.

It is well established that areas LIP and FEF are involved in saccadic eye movements and the visuospatial guidance of eye movements and attention (Bisley and Goldberg, 2010; Buschman and Kastner, 2015). We refer to this network configuration as a visuo-saccadic network. We believe this network requires higher-level visual object processes and motion analyses (e.g., V4 and MT/MST). Additionally, the network involves extracting and guiding the visuospatial coordination of eye movements (e.g., LIP and FEF) during free-viewing. Single-unit studies of macaque FEF during natural visual search indicated that FEF activity contributes to top-down selection (Glaser et al., 2020; Juan et al., 2004; Phillips and Segraves, 2010; Ramkumar et al., 2016). On the other hand, neurons in area V4 converge bottom-up salient visual information with a top-down selection of eye movements for target locations (Mazer and Gallant, 2003; Zhou and Desimone, 2011). Moreover, similar visuo-saccadic networks were also reported in marmosets and humans during free-viewing (Schaeffer et al., 2019), indicating a conservation of this network across primate species.

Overall, our findings suggest that free-viewing networks captured a large part of the underlying anatomical and functional architecture (Kaiser and Hilgetag, 2006; McMahon et al., 2015). Moreover, the robustness of the visuo-saccadic network and the high feasibility of detecting it during free-viewing (e.g., no task-based training inside the scanner) makes it an ideal network for studying dysfunctional changes in patients with Parkinson's (Fukushima et al., 1994) and other neurodegenerative diseases (Antoniades and Kennard, 2015) often expressing deficits in saccadic function.

### 4.2 Imperfect relationship between structure and function in NHP brain networks

The relationship between structure and function in macroscale brain networks is a critical step for developing large-scale neural simulations and informative models of brain function (Shen et al., 2019). Our initial work here demonstrates the feasibility of obtaining meaningful structural and functional graphs derived from awake NHP neuroimaging data with multiple features showing well known brain networks properties.

From our comparative networks analyses, we observed differential connectivity architecture between structural and free-viewing networks. Structural networks showed higher intrahemispheric connectivity than free-viewing networks, which rather showed higher interhemispheric connectivity. This differential architecture is in close correspondence with human macroscale network studies showing greater intrahemispheric density in DTI-based networks as compared to functional brain networks (Bonilha et al., 2015). The direct comparisons between networks show only a positive moderate correlation in correspondence with the relationship between function and structure in human brain networks (Honey et al., 2009; Suárez et al., 2020). Moreover, when we derived visual subnetworks from matching regional parcellations (n regions = 12), we observed a closer relationship between free-viewing and anatomical networks (either track-tracing or structural networks derived from DTI). We believe that these results generally reflect the dominance of the visual sub-network in the Markov study and on the fact that during free-viewing an increased functional connectivity is to be expected. Important to consider in our comparisons, is the disadvantage that relates to the unavailability of standard tract-tracing datasets independent of brain atlases. Thus, our results across networks are rather limited to the matching nomenclature across different brain atlases (*n* = 12) which is a small sample of the larger macro structure. In the future, the availability of track-tracing datasets independent of atlas type will benefit comparisons across macaque brain networks and domains.

When we expanded our comparisons to path length distributions between free-viewing and structural networks we found an increase in path length in free-viewing networks. The path length distribution of structural networks is consistent with the interareal distances described for the cerebral cortex of multiple mamallian species (Song et al., 2014), including those derived from macaque tract-tracing data (Markov et al., 2014). An important biological facet to consider for path length measures in structural networks is the fact that connections are physically constrained by the embedded brain architecture which carries a metabolic cost, decreasing the probability of finding long-range connections (Horvát et al., 2016). This feature is additionally evident from the local clustering and the hierarchical modular structure and segregation in the structural networks. In contrast, free-viewing networks showed an increase in the number of highly weighted nodes with long path length distribution. These long-distance connections might result from synchronous time-series among poly-synaptic distal regions suggesting that during free-viewing functional interactions might be less distance-dependent than structural connections (Bettinardi et al., 2017; Stisoand Bassett, 2018). However, it is important to consider the fact that path length in correlation-based functional networks are susceptible to interpretation difficulties, given that a strong correlation does not necessarily imply the existence of anatomical connection. Path length measures in functional networks represent sequences of statistical associations (Rubinov and Sporns, 2010), and might not necessarily represent an actual route of information flow.

### 4.3 Advantages, limitations and future directions for free-viewing networks

In primates, most macroscale functional mapping studies have focused on the existence of intrinsic functional networks that show similar properties as those found in humans’ resting-state networks (Vincent et al., 2007). Resting-state networks are, however, highly variable across individuals and evolving periods of an imaging session (Hutchison et al., 2013; Nikolaou et al., 2016; Preti et al., 2017). As a result, under resting-state conditions, it is very challenging to predict the internal states that could trigger dynamical shifts in functional connectivity across any given time window and individual.

Naturalistic imaging in NHPs provides similar advantages as those previously demonstrated in human neuroimaging studies (Eickhoff et al., 2020). These advantages include: (1) the ability to relate network structure with the external stimulation (e.g., movie) sequence paradigm. (2) the ability to drive distal and higher-order regions within the network (e.g., cognitive states). (3) the ability to induce reliable activation patterns across individuals (e.g., high intersubject correlations). (4) increases in contrast-to-noise ratio (e.g., improve data quality). Furthermore, naturalistic imaging engages NHPs during movie watching, possibly comforting animals during scanning periods, resulting in less movement artifacts. In our data, we experienced minimal motion deviations (e.g., shifts below 0.5 mm and rotations below 0.5°), which resulted in a practical motion-free time series. Furthermore, the activation patterns we obtained across subjects were reliable and highlighted the effectiveness of the naturalistic imaging approach. Finally, the fact that we obtained our data within two repetitions of a five-minute scan demonstrates the high contrast to noise ratio available in our dataset, additionally evident from the temporal SNR analyses.

Most importantly, we capture network dynamics with distinct thalamocortical and cortico-cortical interactions present in more ecological states than trial-by-trial designs. Crucially, we point out that while we linked network interactions with the movie's content, our study is limited by the epoch of presentation rather than by the evolving structural temporal motifs in networks (Sizemore and Bassett, 2018). Another limitation relates to the variation of movie content that we presented. It is conceivable that a broader range of scene content might have further refined the implicated network patterns. For example, Hasson et al., 2008 showed that higher-level regions (such as FEF, TPJ, and STS) showed disruptions in the temporal structure during scramble scenes while motion-sensitive areas (MT+) were insensitive to disruptions in the time domain. An interesting future approach for capturing network dynamics might be to induce gradual changes in scene content (either forward or backward) as a strategy for shifting the degree of macroscale interactions.

Another avenue of interest for exploiting the dynamics of naturalistic networks lies in the simultaneous recording of eye movements. The study by Russ et al., 2016 specifically quantified the effects of saccadic eye movements on the functional activation maps utilizing regression analysis (Russ et al., 2016). Their study demonstrated that eye-movement variation resulted in very similar eye-movement patterns across the same movie scene repetitions. From our data, we expect that differential eye movement patterns could have contributed to the network states we observed during the more ecologically valid conditions than during a more controlled fixation-based task. On a more technical note, we sampled functional data at a 1.5 sec per scan, limiting our ability to infer network effects from multiple instances of saccades events occurring during a movie viewing epoch.

Moreover, eye-movement recordings were not always successful on all our animals, limiting our ability to relate brain networks with intrinsic eye-movement data. In the future, it will be of interest to further explore the link between network dynamics and eye movements, including pupil dilation, which is known to cause fluctuations in arousal states that involve cholinergic modulation of large-circuits (Shine et al., 2016). Together with the application of more advanced network analyses, it will further aid the detection of recurrent temporal network states (Bassett et al., 2011) during free-viewing in NHPs.

Given the expansion of graph-theoretical tools in the neurosciences, a robust computational framework has emerged in human neuroimaging (Bassett and Sporns, 2017; Bullmore and Sporns, 2009), which has led to dramatic advances in our understanding of the principles guiding the organization of macroscale brain networks. In our study in NHPs, we believe that new analytical network strategies will enable disentangling spatiotemporal dynamics emerging from repeatable states during cognitive processing. The implementation of multimodal neuroimaging with neurophysiological recordings in NHPs will be critical for characterizing the underlying network principles available in brain networks. In the future, the use of network analysis tools in combination with optogenetic (Klein et al., 2016; Ortiz-Rios et al., 2021; Tervo et al., 2016) or chemogenetic interrogation techniques (Inoue et al., 2015; Oyama et al., 2021) might enable the targeted control of neural circuits via intervention of central nodes, possibly affecting global network states.

## Code Accessibility

Imaging data reported in this paper have been shared (DOI: 10.5281/zenodo.5026036 https://zenodo.org/search?page=1&size=20&q=5026036) and are additionally available in https://github.com/ortizriosm/natural-vision-connectivity along with pre-processing data scripts https://github.com/ortizriosm/natural-vision-connectivity/ In the SchmidLab websites there is additional methodological information about non-human primate research: (https://research.ncl.ac.uk/schmidlab/), (https://research.ncl.ac.uk/schmidlab/). On the Dynamic Connectome Lab (https://www.dynamic-connectome.org), there is further information about brain connectivity.

## CRediT authorship contribution statement

**Michael Ortiz-Rios:** Conceptualization, Methodology, Investigation, Formal analysis, Writing – original draft, Writing – review & editing. **Fabien Balezeau:** Conceptualization, Methodology, Investigation. **Marcus Haag:** Investigation, Formal analysis. **Michael C. Schmid:** Supervision, Writing – review & editing, Funding acquisition. **Marcus Kaiser:** Supervision, Writing – review & editing.

## Declaration of Competing Interest

The authors declare no competing financial interest.

## Appendix Supplementary materials

Image, application 1

Image, application 2
