# Supplementary material for: Dynamic reconfiguration of macaque brain networks during natural vision
Source: Neuroimage. 2021 Dec 1;244:118615. doi: 10.1016/j.neuroimage.2021.118615 (PMC8591371; doi:10.1016/j.neuroimage.2021.118615)

**Supplementary figure legends**

**Suppl. Fig. 1.** **Flow chart for the acquisition and analysis of structural and free-viewing networks.**

**A**. Workflow for the acquisition of structural T1, T2, DWI, and fMRI datasets. All data was approximately acquired within less than two hours of scanning time. **B**. Data pre-processing flow chart for the construction of Structural networks. **C**. Data pre-processing flow chart for the construction of free-viewing networks. We acquired three imaging datasets within a single experimental session: Diffusion-weighted imaging data (DWI, green), anatomical (T1, blue), and echo-planar imaging data (EPI, red). The color-coded arrows show the pre-processing steps for each type of dataset as follows: (*1) Warping the anatomical data into the D99 template to generate an in-session subject surrogate brain; (2) White matter segmentation and surface reconstruction; (3) Atlas parcellation aligned to the In-session anatomy and grey matter segmentation; (4) Rendering of ROIs; (5) DWI standard pre-processing; (6) Diffusion tractography and rendering of streamline tracts; (7) Structural networks construction based on the pairwise number of streamline connections; (8) Standard fMRI pre-processing; (9) GLM and coherence analyses; (10) Wavelet decomposition (frequencies 0.04 - 0.16 Hz); (11) Free-viewing networks construction from pairwise cross-correlation of the mean time series of each ROI.* See the **Methods** section for a detailed description of each step. **BOLD response and fractional anisotropy maps**

**A**. Example echo-planar image shows the overall activation (T-value colormap range 2.3 < 10, FDR corr. q < 0.05) to the movie viewing of each subject. **B**. Example image from each NHP shows the directionally colored fractional anisotropy (FA).

**Suppl. Fig. 2.** **BOLD response and fractional anisotropy maps**

**A**. Example echo-planar image shows the overall activation (T-value colormap range 2.3 < 10, FDR corr. q < 0.05) to the movie viewing of each subject. **B**. Example image from each NHP shows the directionally colored fractional anisotropy (FA).

**Suppl. Fig. 3.** **Independent component analyses and free-viewing pattern**

**A**. Example slices of monkey AL showing the first ICA component pattern (10.33 % of explained variance, 5.68 % of total variance) of example run (5 mins duration). The independent component was observed on the first component of each ICA analysis. The plot below shows the independent-component time course which largely reflects the stimulation rate of 30 secs ON and 30 secs OFF. **B**. Similar plots for monkey DP (9.41 % of explained variance, 5.25 % of total variance), **C** monkey FL (9.04 % of explained variance, 4.62 % of total variance), and **D** monkey VL (14.25 % of explained variance, 6.48 % of total variance). Each ICA map was thresholded at a p-value of < 0.05.

**Suppl. Fig. 4.** **Temporal SNR maps of gray matter for each monkey**

**A**. Four example slices of the temporal SNR maps for each monkey AL, DP, FL, and VL. TSNR values clipped at 100 to ease visual inspection of maps across subjects.

**Suppl. Fig. 5. Wavelet decomposition of BOLD time series.**

**A**. Example time series with four runs concatenated using the maximal overlap discrete wavelet transform (MODWT) with the orthogonal Daubechies wavelet which resulted in 5 levels decompositions ranging from 0.25 to 0.001 Hz. For the construction of the free-viewing matrices, we concentrated on the relatively low-frequency range of 6 to 24 seconds, levels 2 (0.124 – 0.06) and level 3 (0.06 – 0.03). **B**. Constructed matrices of each wavelet Level 2 and Level 5 and their composites L2 and L3 and L4 and L5. The correlation coefficient (r) is between the level shown and the original matrix data without wavelet decomposition.

**Suppl. Fig. 6**. **Axial views of functional** **maps during free-viewing and example visual bundle tracts from the optic radiation.**

**A**. Block design paradigm with example frames of each movie segment. Movie segments lasted for 30 seconds, followed by 30 seconds of darkness. **B**. Time course (mean ± SEM) of MT voxels from NHP DP showing BOLD responses to each movie segment. Gray shading represents dark periods within an imaging run. The bottom panel shows the Fourier transform of a voxel showing the BOLD response peak at the stimulation rate (0.016 Hz = 1 cycle/60 s). To calculate coherence, we used the voxel peak response and the stimulation rate. **C**. Axial views of coherence maps rendered into a semi-inflated brain surface of each subject. Across all NHPs, regions with coherence modulation (> 0.35) included regions in frontal, parietal, visual, and higher-level visual regions along the inferotemporal gyrus, among others. Regions with significant modulation (coherence > 0.35) included regions in the ventrolateral lateral prefrontal cortex (vlPFC), frontal eye fields (FEF), lateral intraparietal area (LIP), visual regions (V1, V2, V3, V4), motion-sensitive regions (MT, MST, FST) and higher-level visual regions along the inferotemporal gyrus (TE, TEm, TPO, TEO, TEpd, IPa) among others. **D**. Example white-matter projections within the occipital cortex shown for each subject. Optic radiation bundle (OR) showing projections tracts from the LGN to the visual cortex (V1).

**Suppl. Fig. 7**. **Lateral views of functional** **maps during free-viewing and example visual bundle tracts from the forceps major and the longitudinal fasciculus.**

**A**. Lateral views of activation maps during natural free-viewing. Coherence maps are mapped into a semi-inflated brain surface of each subject. Regions with significant modulation (coherence > 0.35) included regions in the ventrolateral lateral prefrontal cortex (vlPFC), frontal eye fields (FEF), lateral intraparietal area (LIP), visual regions (V1, V2, V3, V4), motion-sensitive regions (MT, MST, FST) and higher-level visual regions along the inferotemporal gyrus (TE, TEm, TPO, TEO, TEpd, IPa) among others. **B**. (Left) Example white-matter projections within the occipital cortex shown for each subject. Forceps major (FM) bundle pathway originating in right V1 and projecting through the forceps major into left V1. (Right) Inferior longitudinal fasciculus (ILF) bundle pathway originating in occipital and posterior dorsal temporal regions and projecting to the inferotemporal cortex (IT). For visualization, the plots showed a brain surface of the left hemisphere and were made semi-transparent.

**Suppl. Fig. 8.** **Free-viewing networks node degree distributions.**

Node degree distribution sample from free-viewing networks of each NHP and hemisphere.

**Suppl. Fig. 9.** **Free-viewing networks path length distributions.**

Path length distribution sample from free-viewing networks of each NHP and hemisphere.

**Suppl. Fig.10.** **SN node degree distributions.**

Node degree distribution sample from structural networks of each NHP and hemisphere.

**Suppl. Fig. 11.** **Structural path length distributions.**

Path length distribution sample from structural networks of each NHP and hemisphere.

**Suppl. Fig. 12.** **Clustering coefficient and characteristic path length**.

**A**. Bar plot of clustering coefficients of structural networks for both the original (blue) and rewired (red) of whole-brain (both hemispheres), left and right hemispheres. The transparency allows for the differential visualization between original and rewired networks. **B**. Same as in A for the characteristic path length of SN. **C**. Bar plot of clustering coefficients for free-viewing networks **D**. Bar plot of characteristic path lengths for free-viewing networks.

**Suppl. Fig. 13.** **Structural and functional matrices organized according to overlapping regions from the macaque tract-tracing matrix.** A. DTI matrix for each NHP organized according to macaque tract-tracing data. B. fMRI-based matrices for each NHP are organized according to tract-tracing data.

**Suppl. Fig. 14.** **Movie segment stimuli used for contrast analyses between original scenes and phase scrambled scenes. A**. Example image frame of each movie clip: Ego-motion, Object, Face, Hand action. The movies were presented for 30 seconds, followed by 15 secs of darkness. Each movie category contained four controls (Optic flow, phase scrambling, saliency contour, and tile scrambling). **B**. GLM contrast (q-FDR < 0.05 corrected) between each scene category and correspondent phase scramble scenes shows activation patterns across the inferotemporal and frontal cortices of monkey DP. For each contrast, the t-value color range (4.8 > t < 8) allows the comparison across the activation magnitude of each scene category. **C**. Same contrast mapping for monkey AL.

**Suppl. Fig. 15.** **Network hubs for each monkey during free-viewing.** Hubs with high eigenvector centrality highlighting regions with high importance in the visual network during natural free-viewing. Hub regions for each monkey show eigenvector centrality (c) (c > 1_x_10^-4^; n highlight for size >= 1 Z-score) highlighting the central nodes during free-viewing.

**Suppl. Fig. 16.** **Modularity of structural networks.**

**A**. Average structural connectivity showing the logarithmic number of streamlines touching every pair of ROIs for both hemispheres. **B**. Circular dendrogram plot with hierarchical edge bundles aiding the visualization of the structural organization color-coding same as in **Fig. 4C**. The node face edge shows node degree connectivity while the edges.


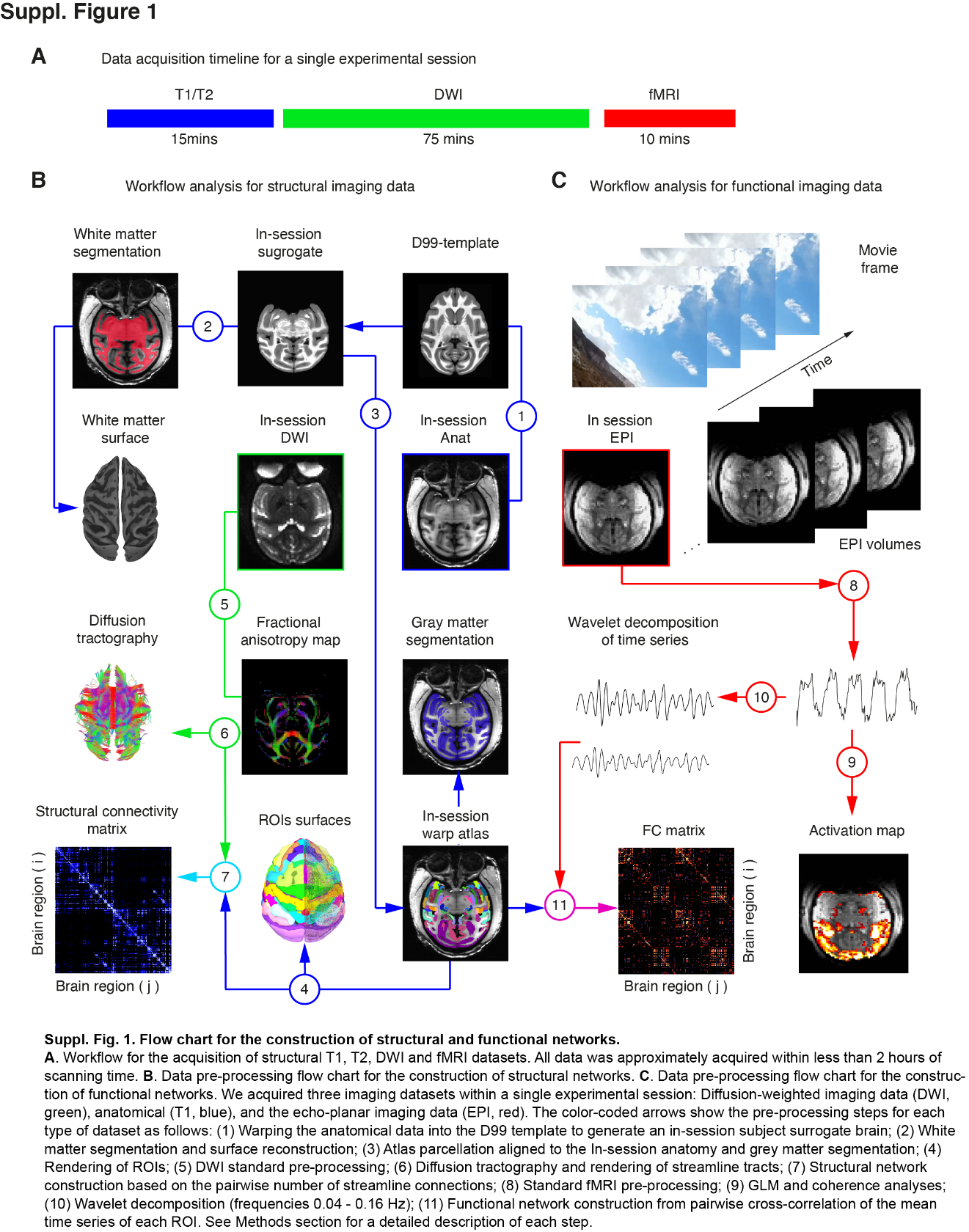


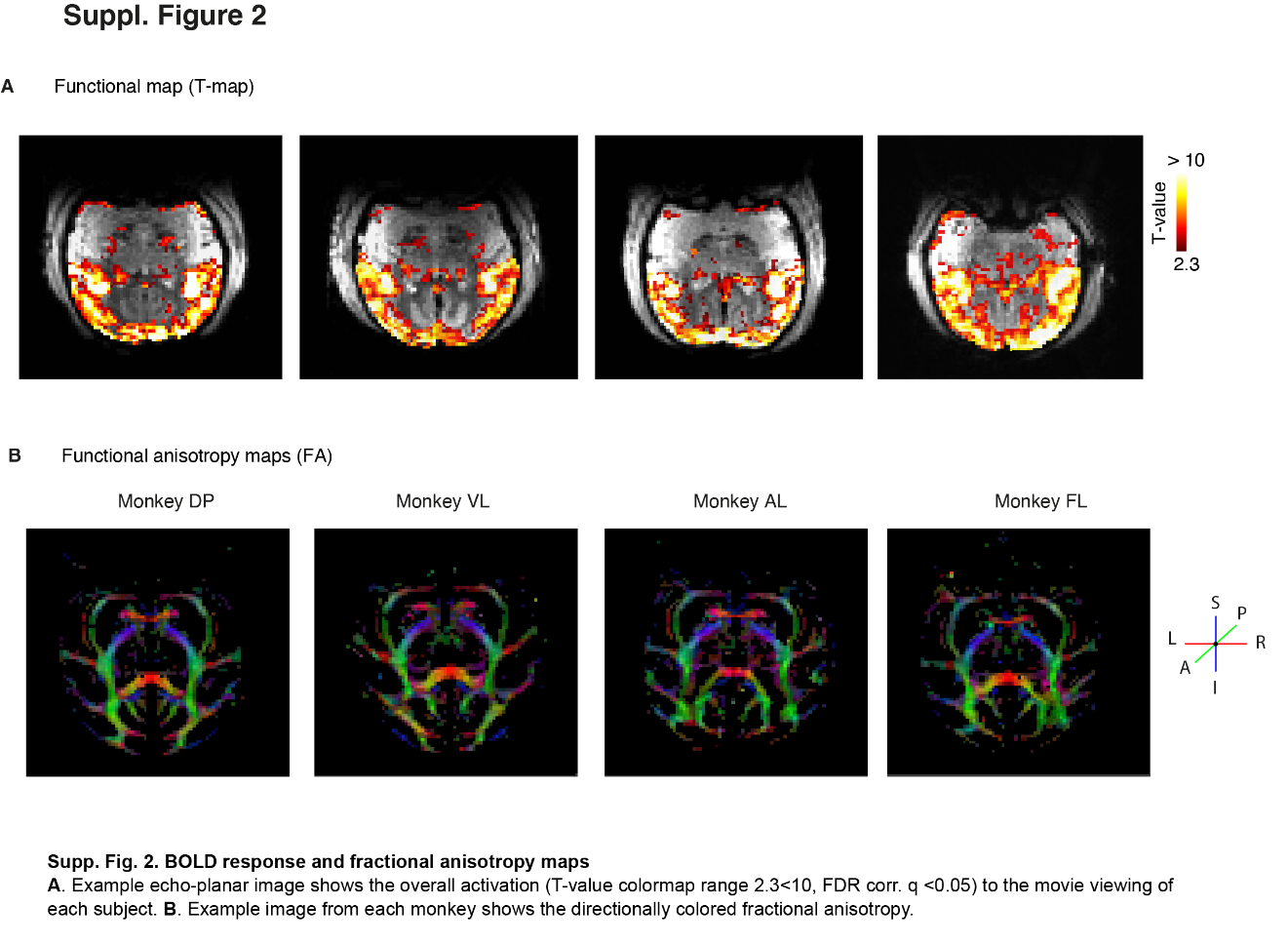


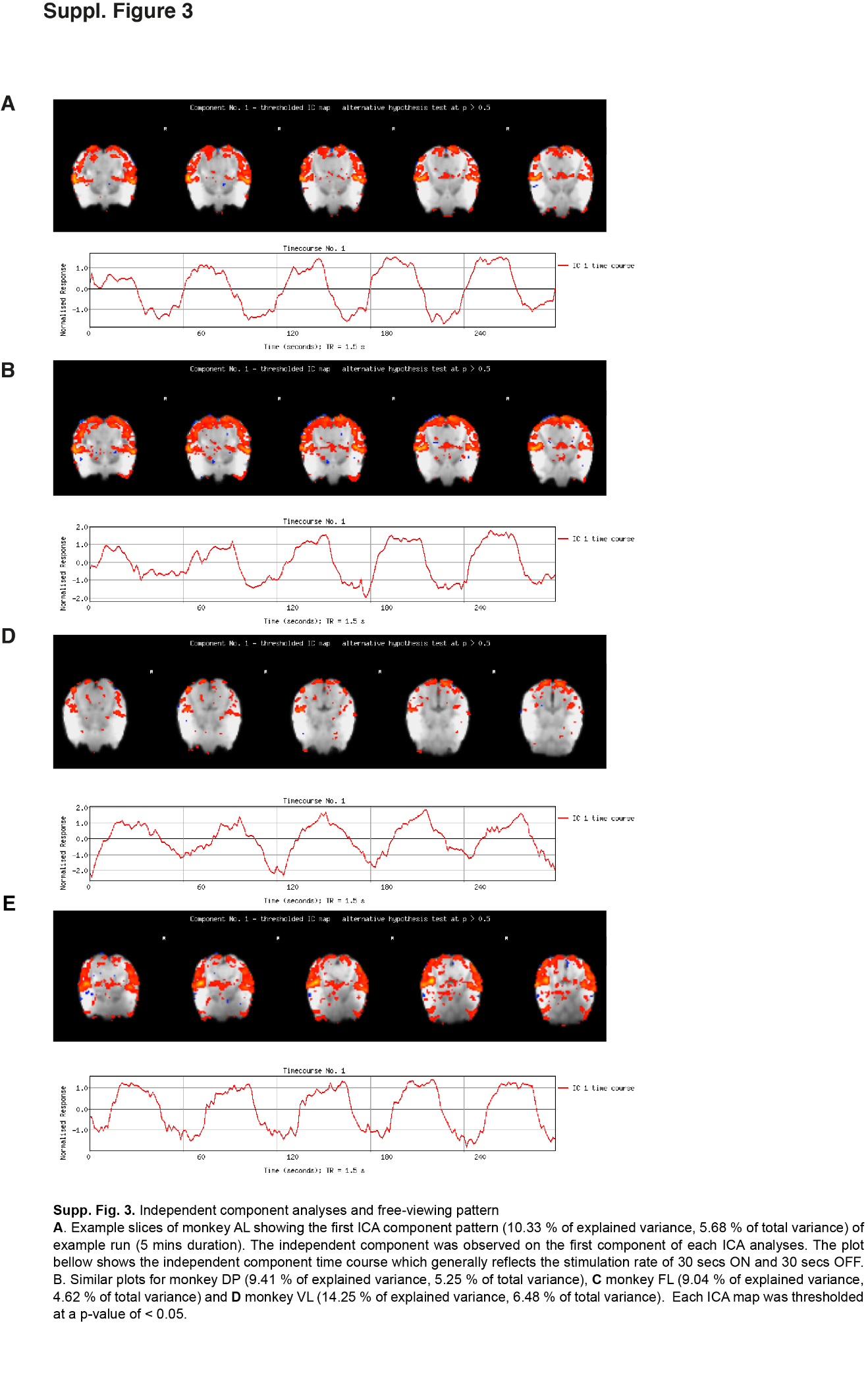


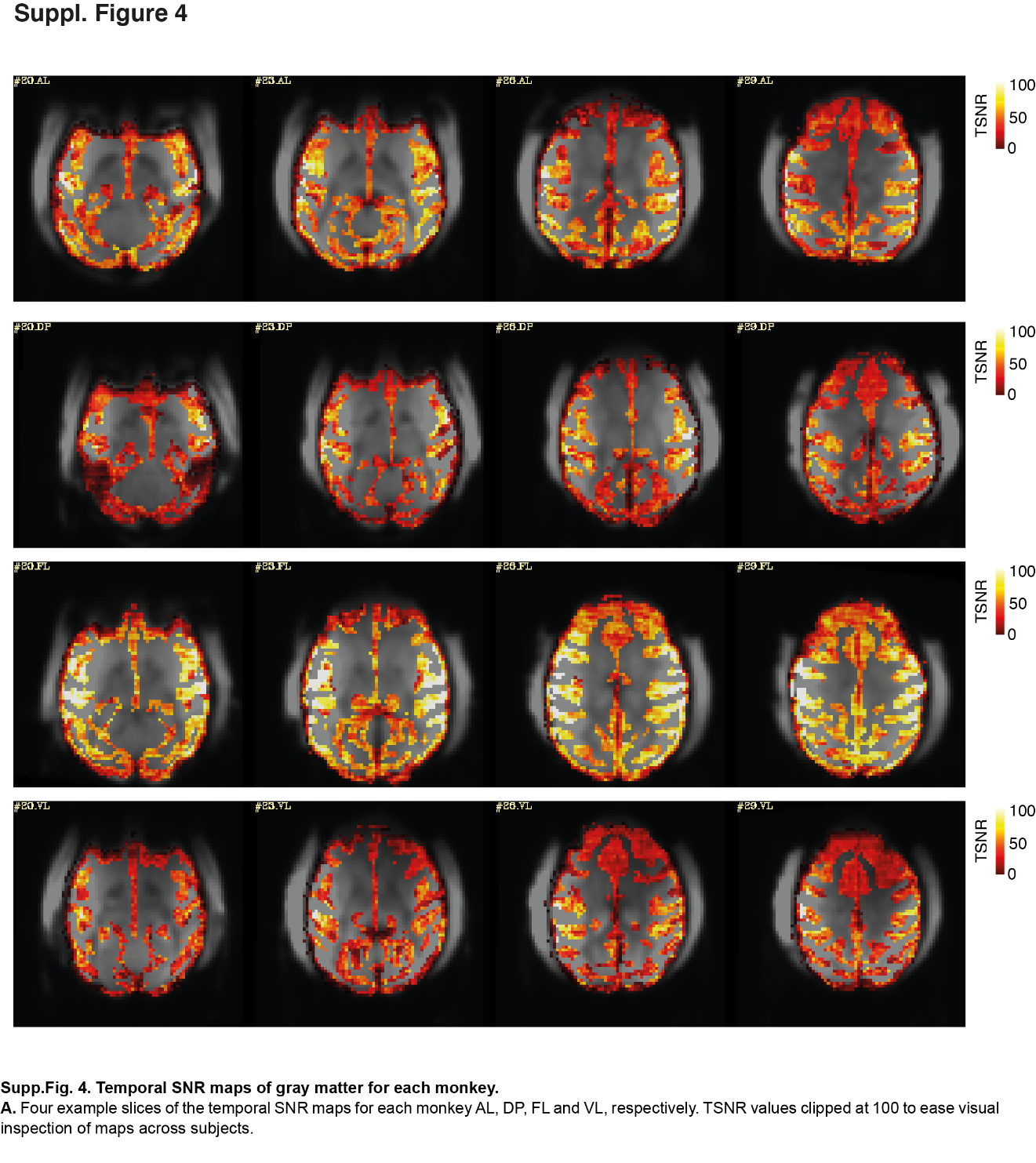


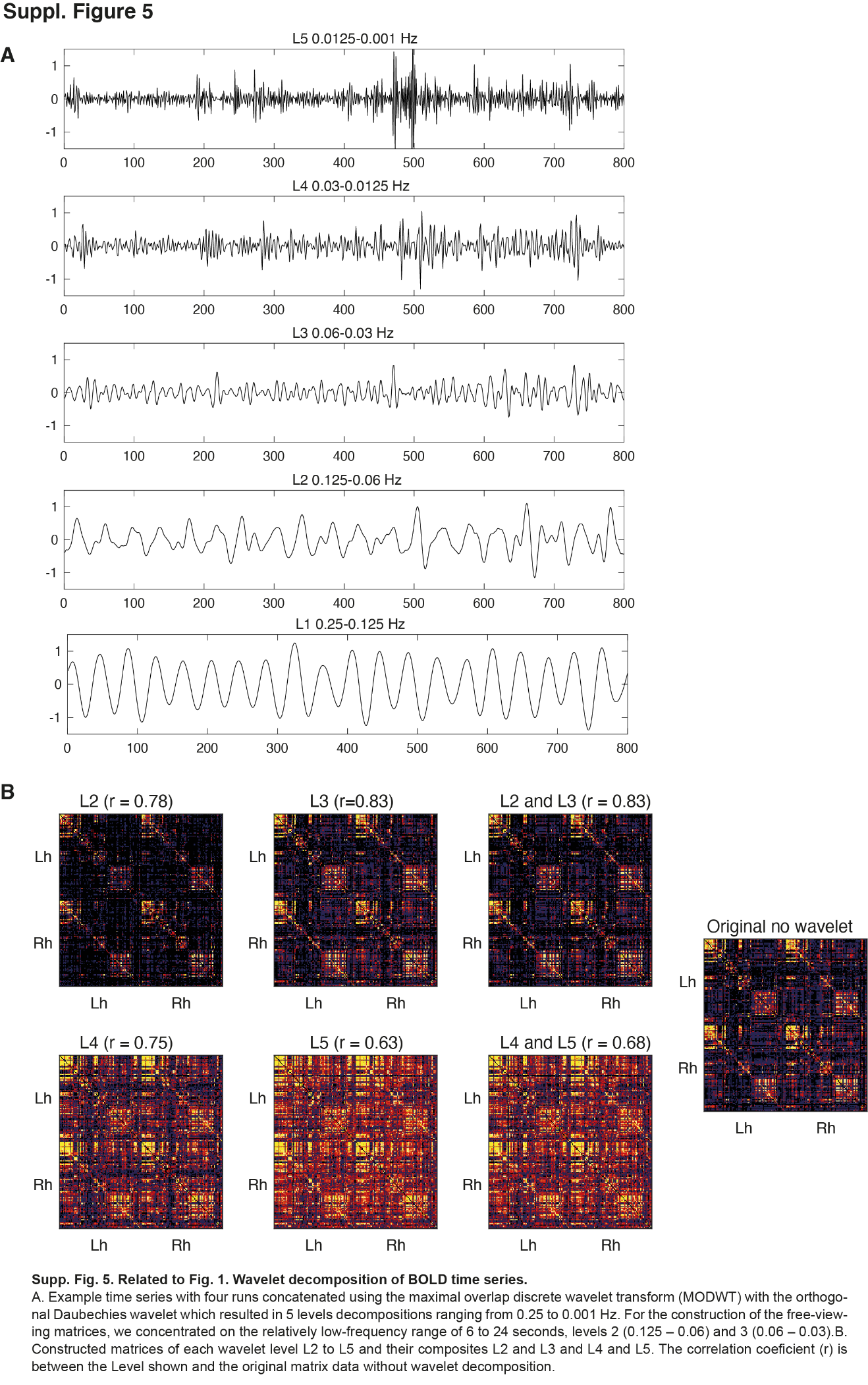


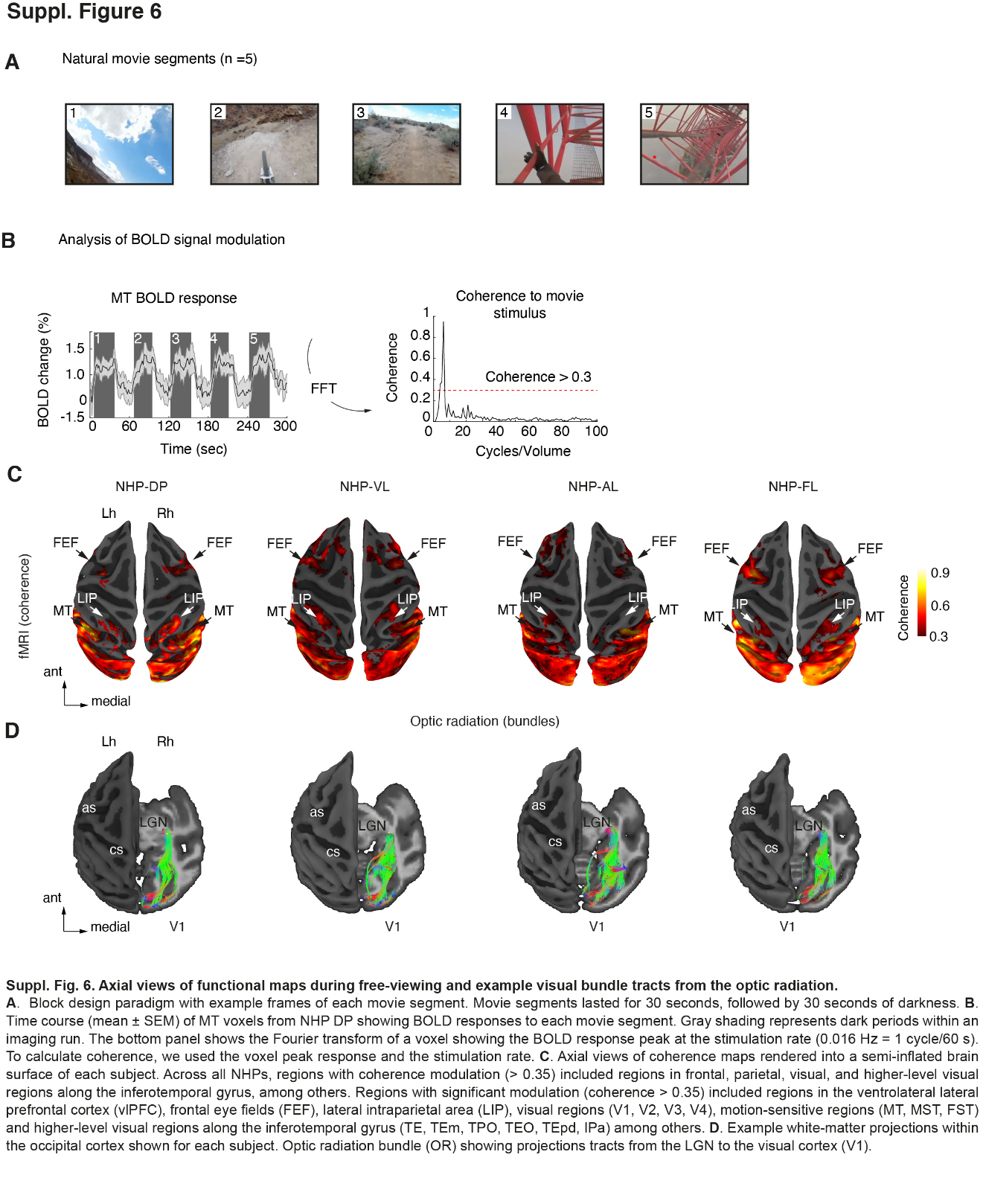


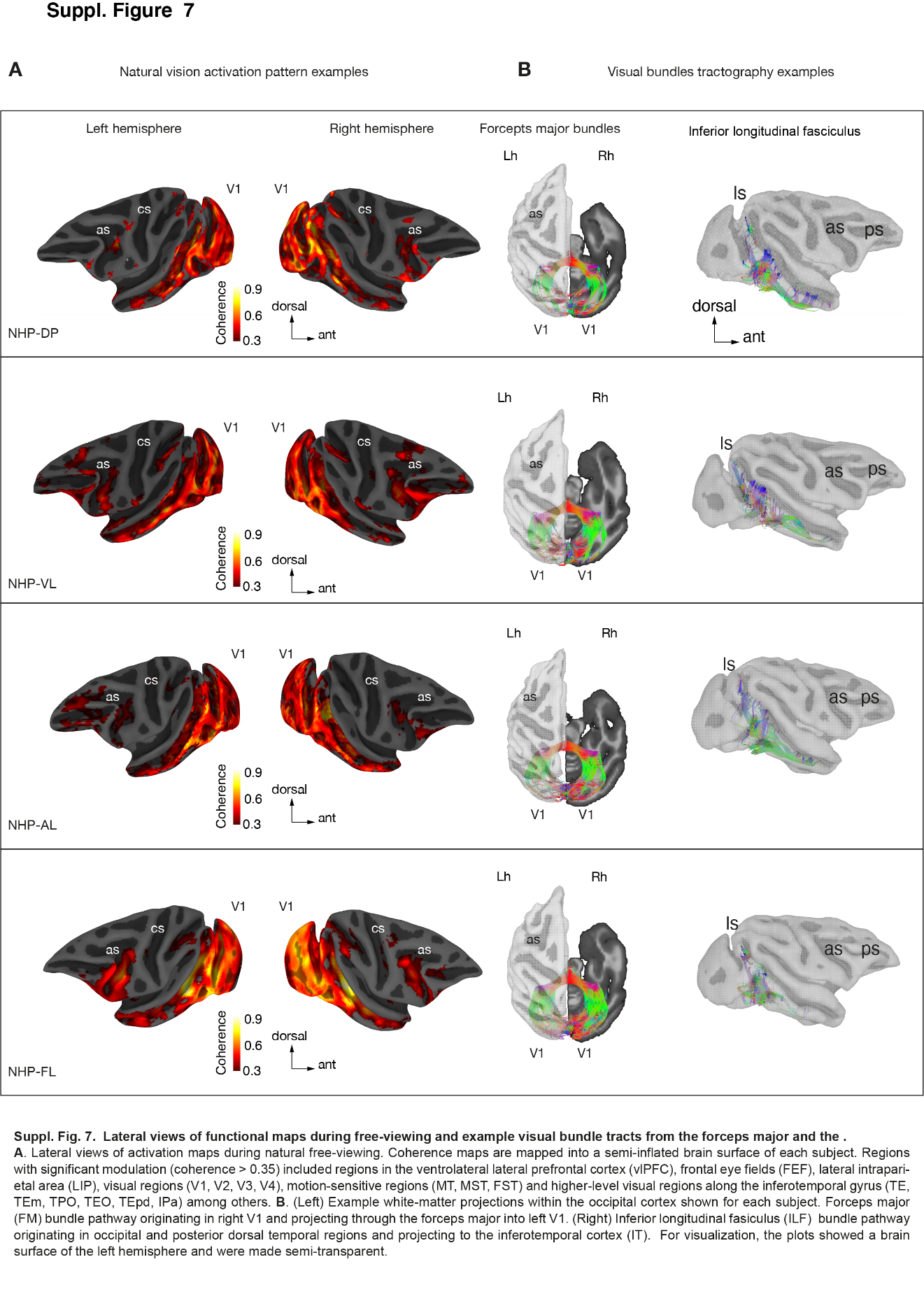


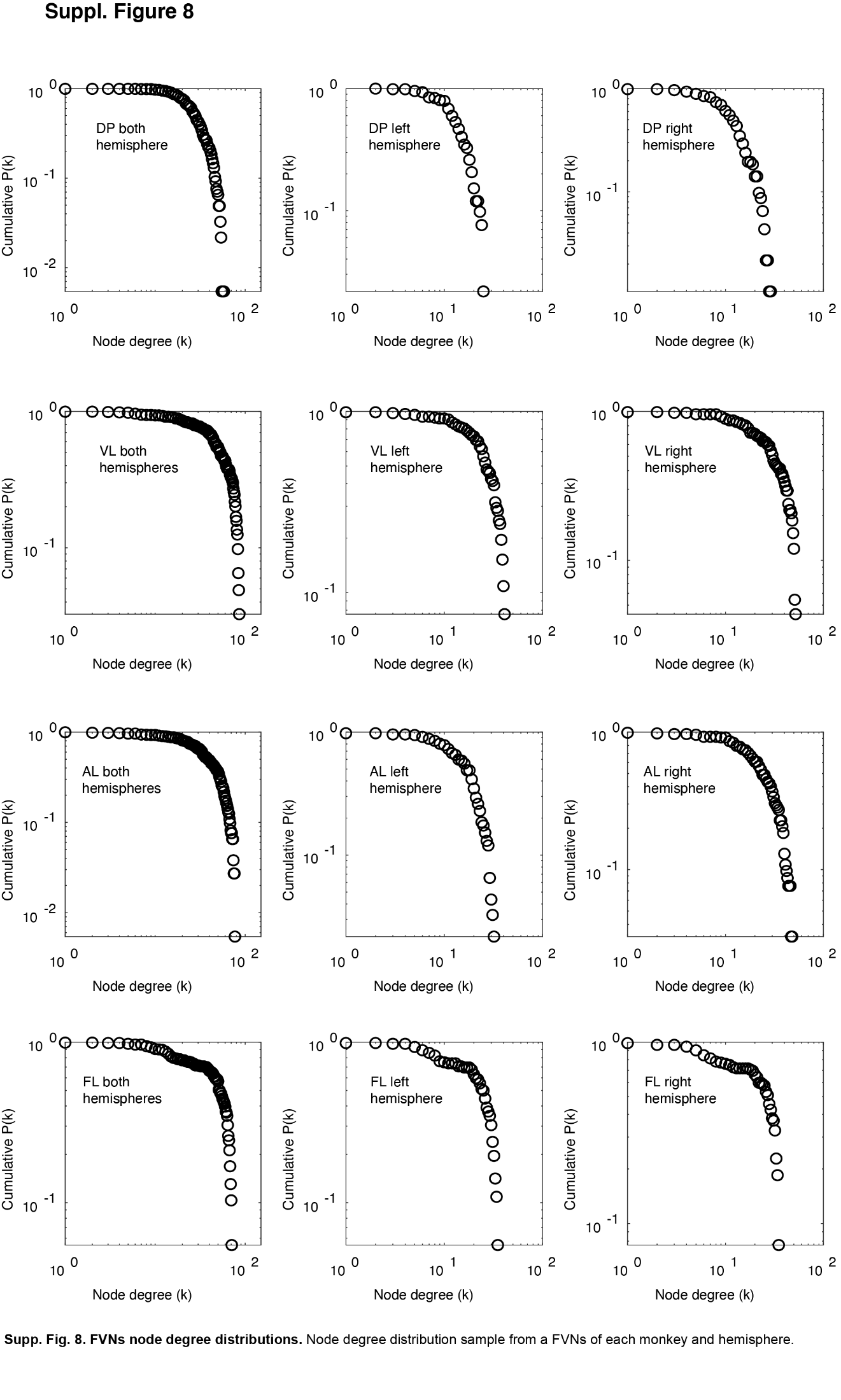


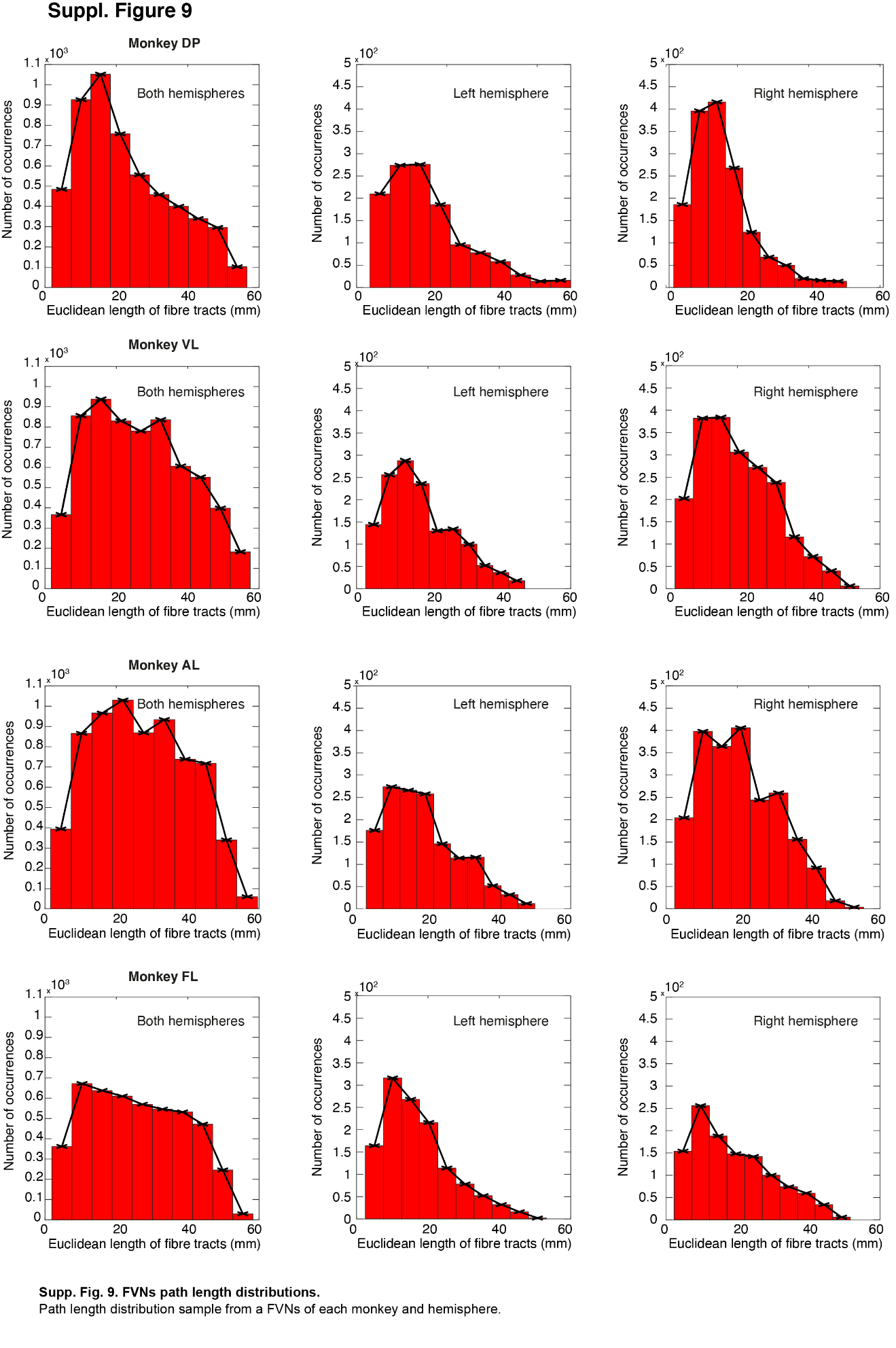


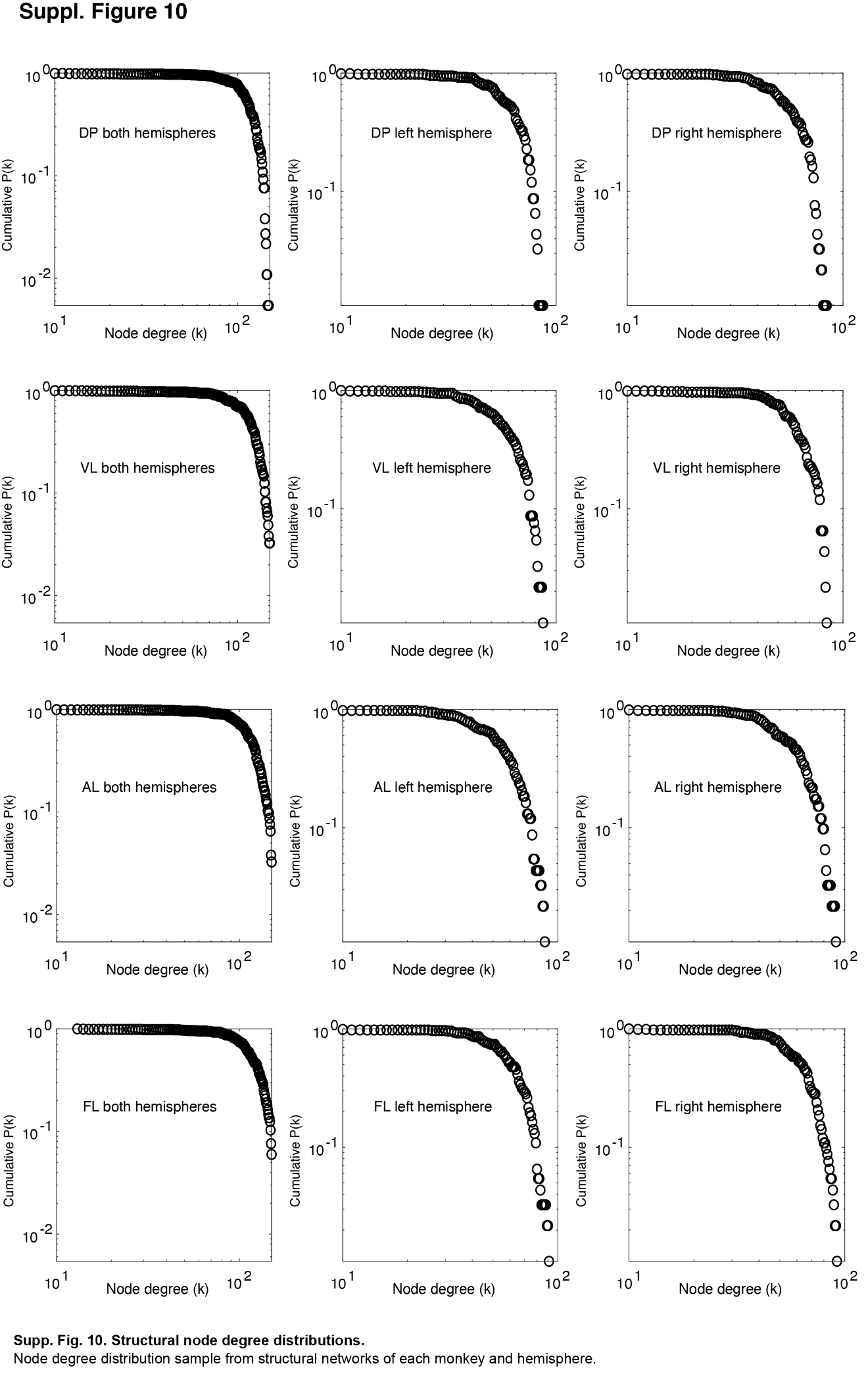


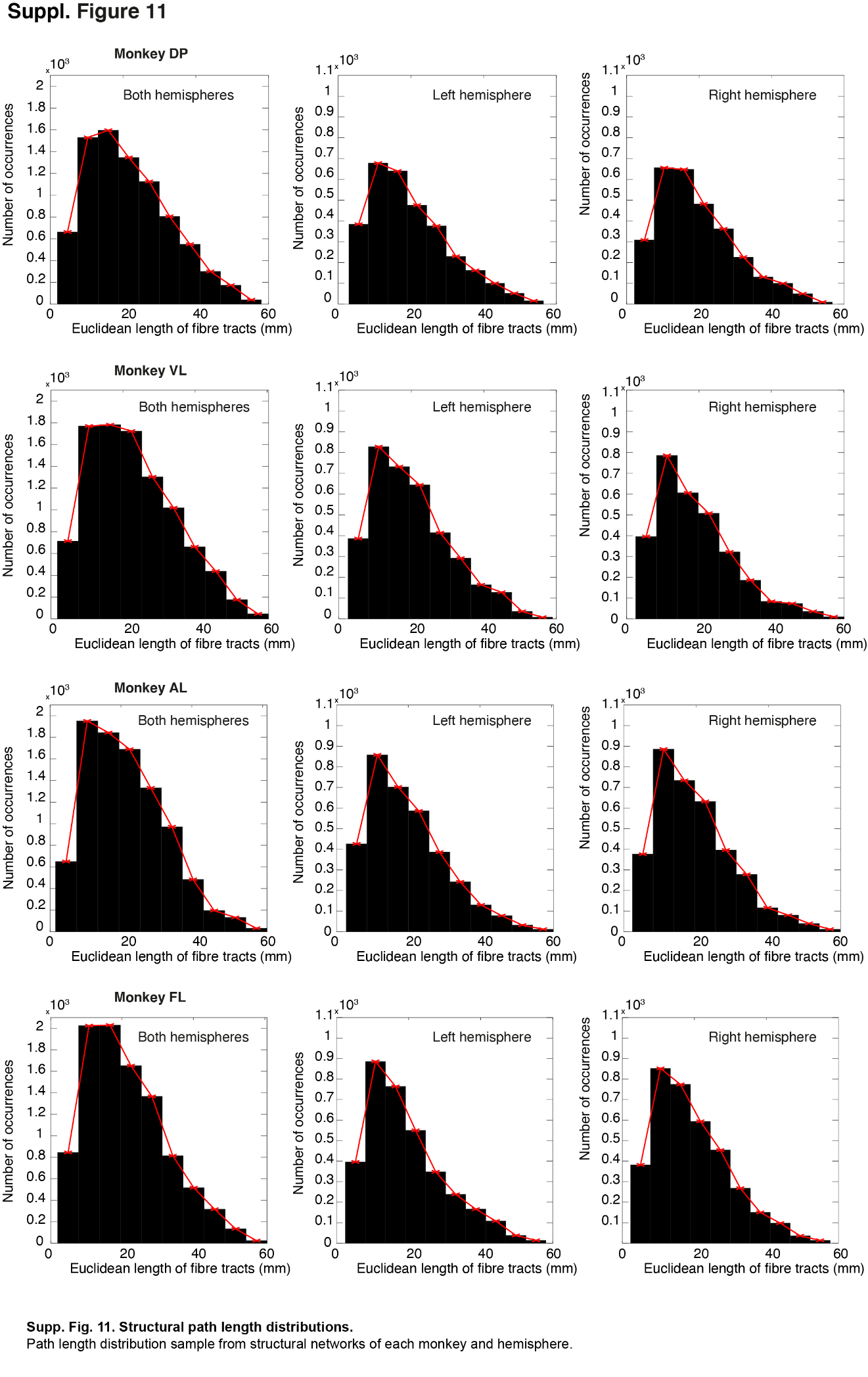


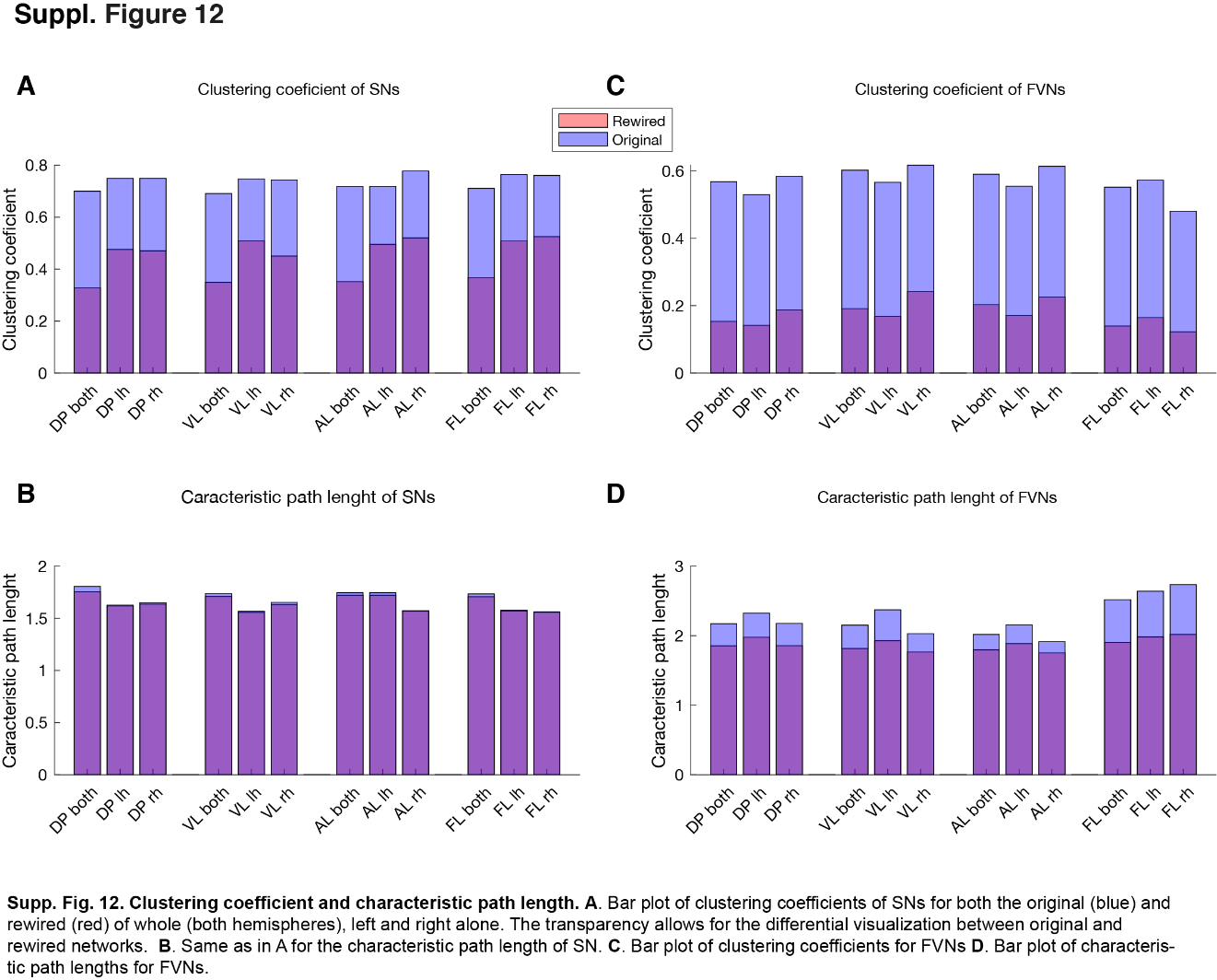

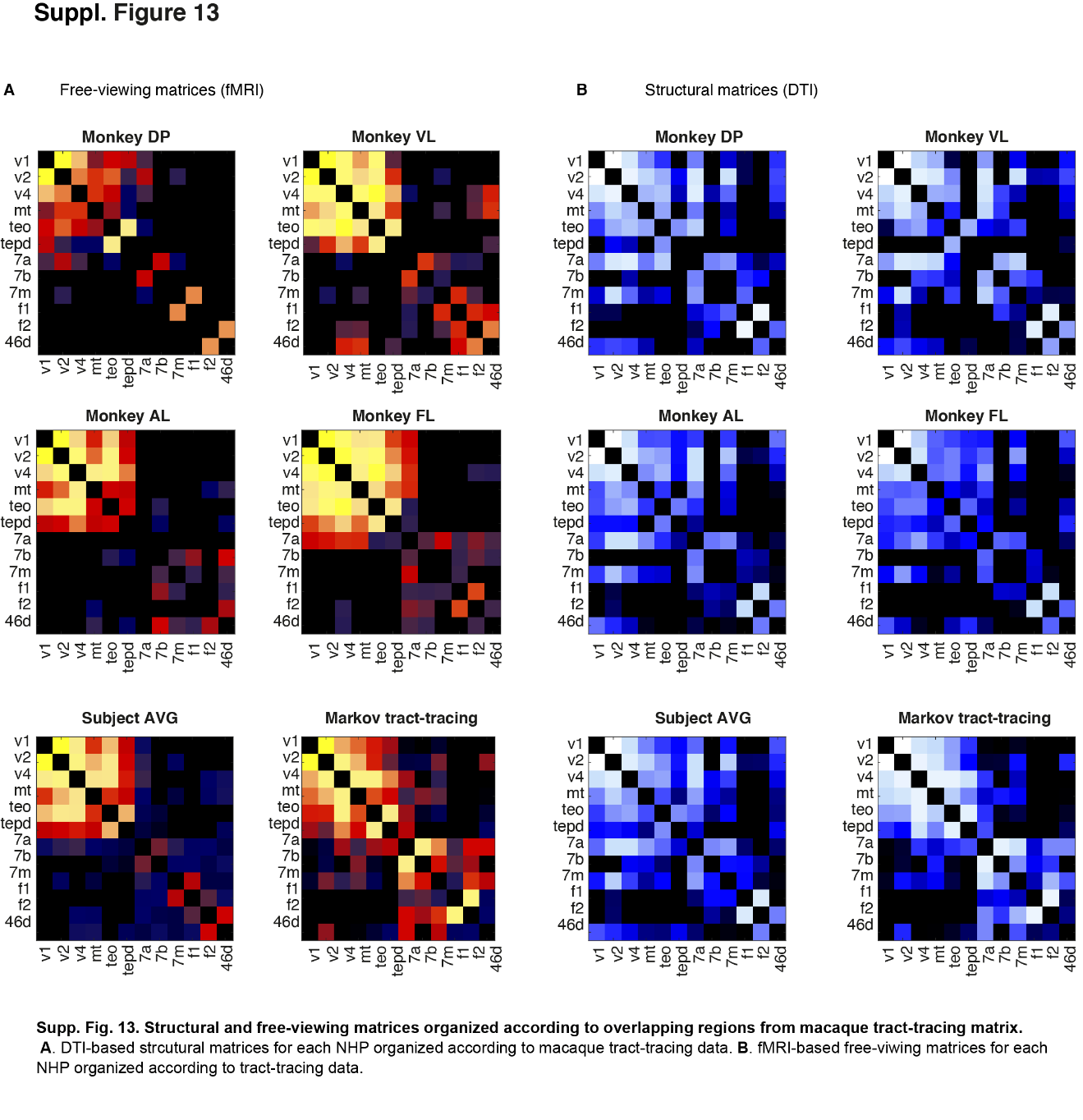

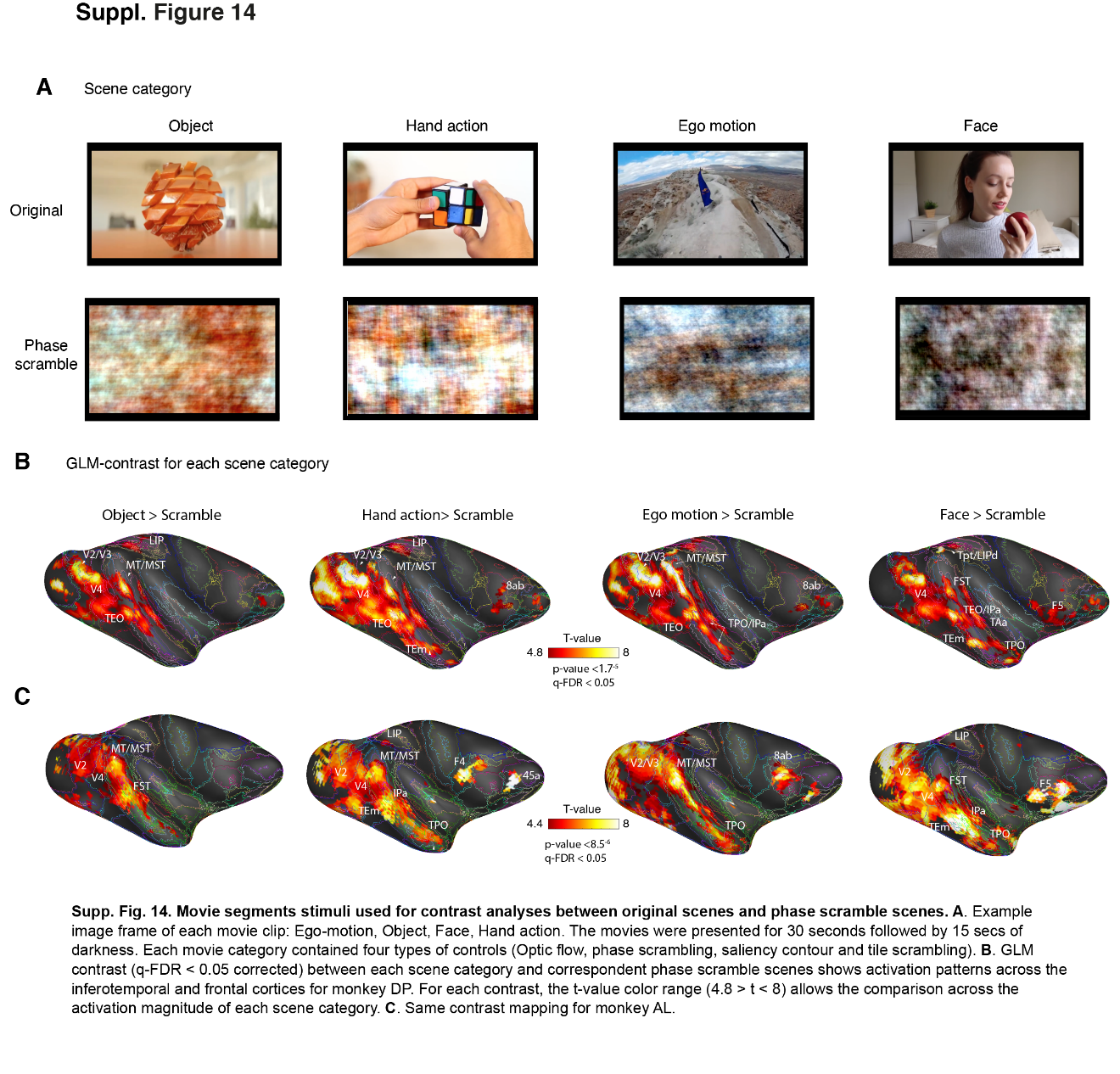

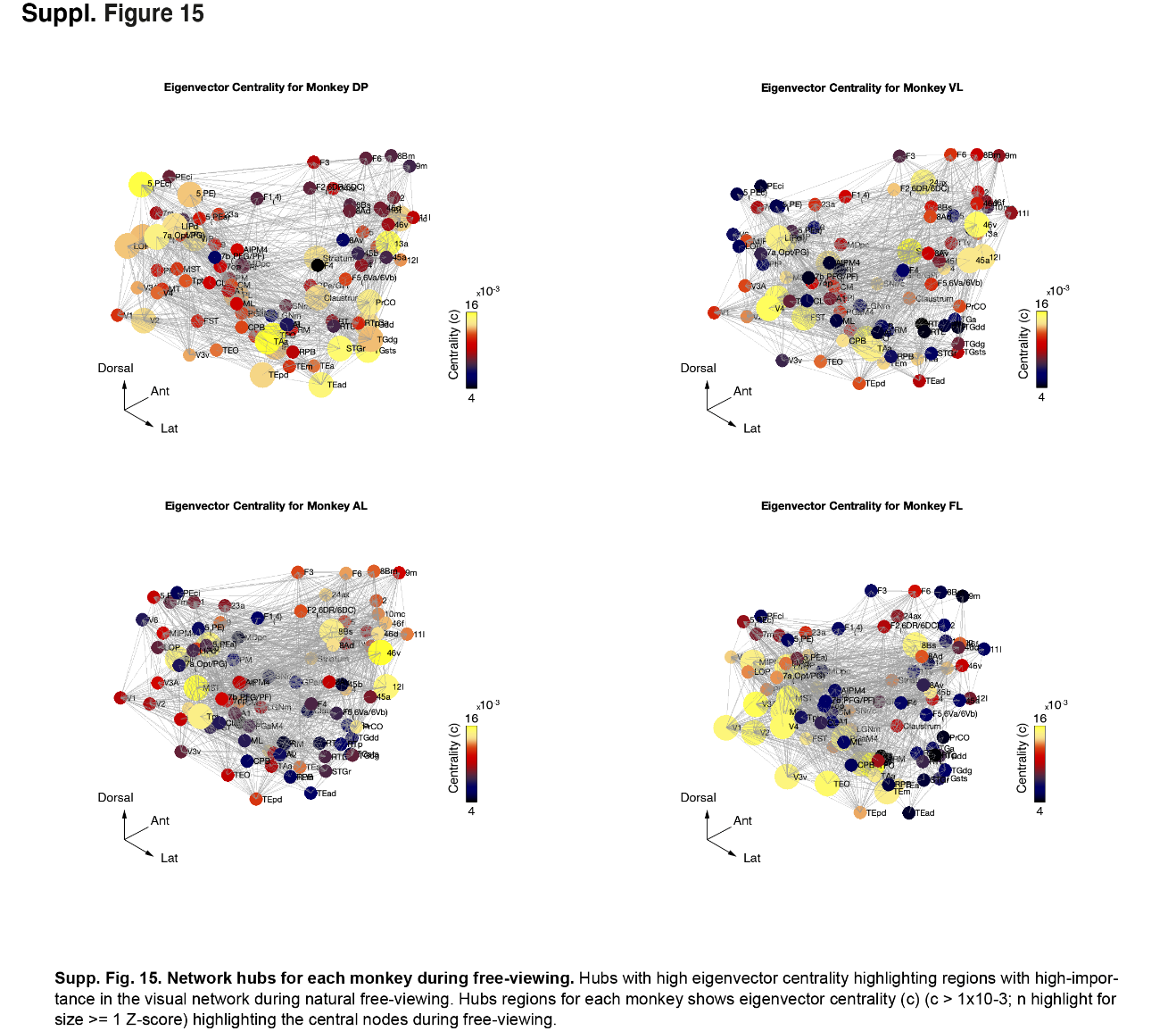


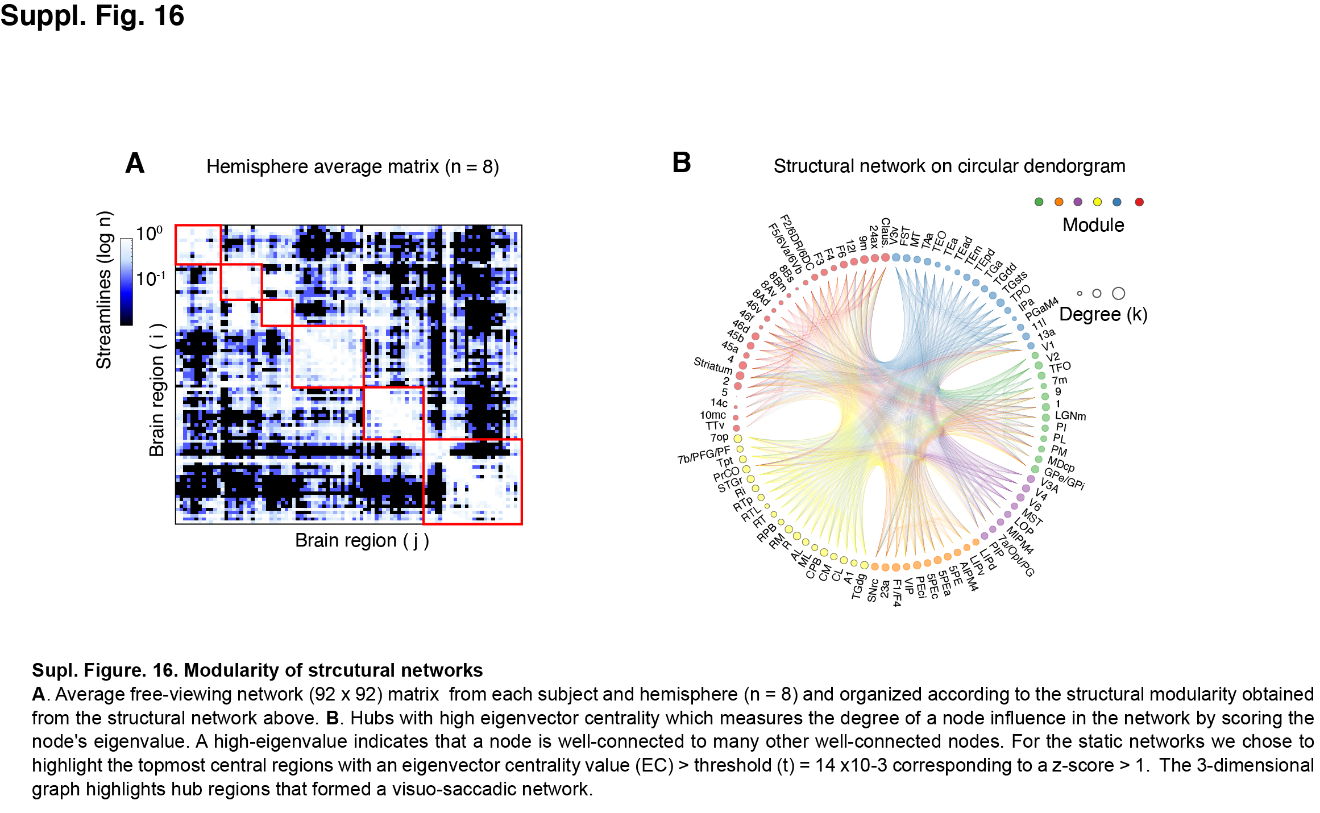

Supplement: Supplementary file 1 [file mmc1.docx]
