# Supplementary material for: Dynamic reconfiguration of macaque brain networks during natural vision
Source: Neuroimage. 2021 Dec 1;244:118615. doi: 10.1016/j.neuroimage.2021.118615 (PMC8591371; doi:10.1016/j.neuroimage.2021.118615)
Supplement: Supplementary file 2 [file mmc2.docx]

**Dynamic reconfiguration of macaque brain networks during natural vision**

**Authors**: Michael Ortiz-Rios^1, 2*^, Fabien Balezeau^1^, Marcus Haag^1, 5^, Michael C. Schmid^1,6^, Marcus Kaiser^3, 4, 5^

**Authors: Affiliations**: ^1^Bioscience Institute, Henry Welcome Building, Medical School, Framlington Place, Newcastle upon Tyne, NE2 4HH, UK; ^2^Functional Imaging Laboratory, Deutsches Primatenzentrum (DPZ), Leibniz-Institut für Primatenforschung, Göttingen, Germany; ^3^ School of Computing, Urban Sciences Building, Newcastle University, Science Central, Newcastle upon Tyne, NE4 5TG, UK; ^4^ Precision Imaging Beacon, School of Medicine, University of Nottingham, UK; ^5^ Shanghai Jiao Tong University, Rui Jin Hospital, Department of Functional Neurosurgery, China; ^6^ Faculty of Science and Medicine, University of Fribourg, Chemin du Musée 5, 1700 Fribourg, Switzerland

**Corresponding author**: *Michael Ortiz-Rios

**Email address:**  [mortiz-rios@dpz.eu](mailto:mortiz-rios@dpz.eu)

|  |  | **DWI** |  |  |  | **EPI** | |  |  | **MDEFT** | |  |
| --- | --- | --- | --- | --- | --- | --- | --- | --- | --- | --- | --- | --- |
| **Subj.Sess.** | **V.Nr1** | **D.Ip1** | **F.O41** | **A.O01** | **V.GL1** | **D.GI2** | **F.MB1** | **A.Pr1** | **V.GL1** | **D.GI2** | **F.MB1** | **A.Pr1** |
| **FA (°)** | 65 | 65 | 65 | 65 | 65 | 65 | 65 | 65 | 90 | 90 | 90 | 90 |
| **TE (ms)** | 58 | 58 | 58 | 58 | 21 | 21 | 21 | 21 | 3.74 | 3.74 | 3.74 | 3.74 |
| **TR (ms)** | 14200 | 14200 | 14200 | 14200 | 1500 | 1500 | 1500 | 1500 | 2000 | 2000 | 2000 | 2000 |
| **BW (Hz/Px)** | 1495 | 1495 | 1495 | 1495 | 1704 | 1704 | 1704 | 1704 | 284 | 284 | 284 | 284 |
| **ES (ms)** | 58 | 58 | 58 | 58 | 58 | 58 | 58 | 58 |  |  |  |  |
| **Acl. Fac.** | 2 | 2 | 2 | 2 | 2 | 2 | 2 | 2 | 1 | 1 | 1 | 1 |
| **Seq.** | Dw-SE-EPI | Dw-SE-EPI | Dw-SE-EPI | Dw-SE-EPI | GE-EPI | GE-EPI | GE-EPI | GE-EPI | MDEFT | MDEFT | MDEFT | MDEFT |
| **B val (s/mm2)** | 800 | 800 | 800 | 800 | x | x | x | x | x | x | x | x |
| **Bo im.** | 4 | 4 | 4 | 4 | x | x | x | x | x | x | x | x |
| **B dir.** | 60 | 60 | 60 | 60 | x | x | x | x | x | x | x | x |
| **RR-RO (mm)** | 0.97 | 0.97 | 0.97 | 0.97 | 1.2 | 1.2 | 1.2 | 1.2 | 0.61 | 0.61 | 0.61 | 0.61 |
| **RR-PH (mm)** | 0.97 | 0.97 | 0.97 | 0.97 | 1.2 | 1.2 | 1.2 | 1.2 | 0.61 | 0.61 | 0.61 | 0.61 |
| **RR-SL (mm)** | 1 | 1 | 1 | 1 | 1.2 | 1.2 | 1.2 | 1.2 | 0.62 | 0.62 | 0.62 | 0.62 |
| **RIM-RO (px)** | 88 | 88 | 88 | 88 | 88 | 88 | 88 | 88 | 176 | 176 | 176 | 176 |
| **RIM-PH (px)** | 88 | 88 | 88 | 88 | 88 | 88 | 88 | 88 | 176 | 176 | 176 | 176 |
| **RIM-SL (px)** | 56 | 56 | 56 | 56 | 31 | 34 | 34 | 34 | 72 | 74 | 96 | 74 |
| **Nacq** | 5 | 5 | 5 | 5 | 200 | 200 | 200 | 200 | 1 | 1 | 1 | 1 |
| **TA (h:m:s)** | 01:15 | 01:15 | 01:15 | 01:15 | 05:00 | 05:00 | 05:00 | 04:24 | 14:24 | 14:48 | 19:12 | 04:24 |

**Supplementary Table 1**. Acquisition parameters for each subject session and of type dataset: DWI, EPI and MDEFT. (Left) a diffusion-weighted spin-echo EPI sequence (DW SE-EPI) sequence was used to acquire DWI images for white matter tractography. (Middle) a gradient-echo (GE) EPI sequence was used to acquire BOLD signal modulation for functional imaging. (Right) a magnetization-prepared RApid gradient echo (MP-RAGE) sequence was used to acquire anatomical (T1) images for cortical white and gray matter segmentation and atlas parcellation. Nacq, (Number of acquisitions); Acl. Fac., (Acceleration factor); Seq. (Sequence), Bo im., (number of B0 images); B dir. (number of B directions).

| Supp.Table2  subj.session.run | component num. | Explained variance | Total variance | Mean GM TSNR |
| --- | --- | --- | --- | --- |
| V.GL1.r1 | 1 | 14.25% | 6.48% | 46.04 |
| V.GL1.r2 | 1 | 12.51% | 6.58% | 43.9 |
| V.GL1.r3 | 1 | 12.51% | 6.33% | 46.83 |
| DP.GI2.r1 | 1 | 9.41% | 5.25% | 46.36 |
| DP.GI2.r2 | 1 | 12.27% | 6.29% | 48.71 |
| DP.GI2.r3 | 1 | 8.40% | 5.04% | 51.37 |
| DP.GI2.r4 | 1 | 7.22%% | 4.24% | 44.69 |
| F.MB1.r1 | 1 | 9.05% | 4.62% | 59.62 |
| A.Pr1.r1 | 1 | 10.33% | 5.68% | 81.99 |
| A.Pr1.r2 | 1 | 9.38% | 5.24% | 57.92 |

**Supplementary Table 2.** Small-world properties of structural brain networks obtained from binarized and rewired networks sharing the same degree distribution. Both, both hemispheres; lh, left hemisphere; rh, right hemisphere.

**Supp. Table 2. Network properties.** Small-world properties of structural brain networks obtained from binarized and rewired networks sharing the same degree distribution. Both, both hemispheres; lh, left hemisphere; rh, right hemisphere.

| **NHP-Hem-Net** | **Edge**  **density** | **Clustering**  **coefficient**  **rewired** | **Clustering coefficient** | | **Characteristic path length, rewired** | | **Characteristic path length** |
| --- | --- | --- | --- | --- | --- | --- | --- |
| DP-both-SN | 0.2469 | 0.3283 | 0.7003 | 1.7550 | | 1.8052 | |
| DP-lh-SN | 0.3829 | 0.4757 | 0.7492 | 1.6190 | | 1.6274 | |
| DP-rh-SN | 0.3660 | 0.4705 | 0.7494 | 1.6362 | | 1.6503 | |
| VL-both-SN  VL-lh-SN | 0.2916  0.4448 | 0.3496  0.5084 | 0.6911  0.7477 | 1.7092  1.5573 | | 1.7385  1.5676 | |
| VL-rh-SN | 0.3703 | 0.4504 | 0.7436 | 1.6321 | | 1.6522 | |
| AL-both-SN  AL-lh-SN | 0.2812  0.4233 | 0.3520  0.4957 | 0.7185  0.7185 | 1.7218  1.7218 | | 1.7461  1.7461 | |
| AL-rh-SN  FL-both-SN  FL-lh-SN  FL-rh-SN | 0.4350  0.2941  0.4300  0.4436 | 0.5205  0.3674  0.5081  0.5250 | 0.7777  0.7112  0.7645  0.7606 | 1.5670  1.7069  1.5714  1.5584 | | 1.5730  1.7342  1.5789  1.5636 | |
| DP-both-FN | 0.1595 | 0.1534 | 0.5683 | 1.8550 | | 2.1729 | |
| DP-lh-FN | 0.1476 | 0.1419 | 0.5297 | 1.9763 | | 2.3239 | |
| DP-rh-FN | 0.1861 | 0.1877 | 0.5844 | 1.8564 | | 2.1770 | |
| VL-both-FN  VL-lh-FN | 0.1884  0.1665 | 0.1913  0.1686 | 0.6023  0.5663 | 1.8164  1.9288 | | 2.1519  2.3725 | |
| VL-rh-FN | 0.2410 | 0.2418 | 0.6169 | 1.7659 | | 2.0293 | |
| AL-both-FN  AL-lh-FN | 0.2053  0.1727 | 0.2028  0.1710 | 0.5904  0.5547 | 1.7978  1.8860 | | 2.0170  2.1571 | |
| AL-rh-FN  FL-both-FN  FL-lh-FN  FL-rh-FN | 0.2563  0.1389  0.1503  0.1388 | 0.2258  0.1397  0.1644  0.1224 | 0.6137  0.5519  0.5727  0.4806 | 1.7518  1.9029  1.9833  2.0177 | | 1.9160  2.5182  2.6407  2.7350 | |
